# Geographic Variations in Arthritis Prevalence, Health-Related Characteristics, and Management — United States, 2015

**DOI:** 10.15585/mmwr.ss6704a1

**Published:** 2018-03-16

**Authors:** Kamil E. Barbour, Susan Moss, Janet B. Croft, Charles G. Helmick, Kristina A. Theis, Teresa J. Brady, Louise B. Murphy, Jennifer M. Hootman, Kurt J. Greenlund, Hua Lu, Yan Wang

**Affiliations:** 1 Division of Population Health, National Center for Chronic Disease Prevention and Health Promotion, CDC, Atlanta, Georgia; 2 G^2^S Corporation, San Antonio, Texas

## Abstract

**Problem/Condition:**

Doctor-diagnosed arthritis is a common chronic condition affecting an estimated 23% (54 million) of adults in the United States, greatly influencing quality of life and costing approximately $300 billion annually. The geographic variations in arthritis prevalence, health-related characteristics, and management among states and territories are unknown. Therefore, public health professionals need to understand arthritis in their areas to target dissemination of evidence-based interventions that reduce arthritis morbidity.

**Reporting Period:**

2015.

**Description of System:**

The Behavioral Risk Factor Surveillance System is an annual, random-digit–dialed landline and cellular telephone survey of noninstitutionalized adults aged ≥18 years residing in the United States. Self-reported data are collected from the 50 states, the District of Columbia, Guam, and Puerto Rico. Unadjusted and age-standardized prevalences of arthritis, arthritis health-related characteristics, and arthritis management were calculated. County-level estimates were calculated using a validated statistical modeling method.

**Results:**

In 2015, in the 50 states and the District of Columbia, median age-standardized prevalence of arthritis was 23.0% (range: 17.2%–33.6%). Modeled prevalence of arthritis varied considerably by county (range: 11.2%–42.7%). In 13 states that administered the arthritis management module, among adults with arthritis, the age-standardized median percentage of participation in a self-management education course was 14.5% (range: 9.1%–19.0%), being told by a health care provider to engage in physical activity or exercise was 58.5% (range: 52.3%–61.9%), and being told to lose weight to manage arthritis symptoms (if overweight or obese) was 44.5% (range: 35.1%–53.2%). Respondents with arthritis who lived in the quartile of states with the highest prevalences of arthritis had the highest percentages of negative health-related characteristics (i.e., arthritis-attributable activity limitations, arthritis-attributable severe joint pain, and arthritis-attributable social participation restriction; ≥14 physically unhealthy days during the past 30 days; ≥14 mentally unhealthy days during the past 30 days; obesity; and leisure-time physical inactivity) and the lowest percentage of leisure-time walking.

**Interpretation:**

The prevalence, health-related characteristics, and management of arthritis varied substantially across states. The modeled prevalence of arthritis varied considerably by county.

**Public Health Action:**

The findings highlight notable geographic variability in prevalence, health-related characteristics, and management of arthritis. Targeted use of evidence-based interventions that focus on physical activity and self-management education can reduce pain and improve function and quality of life for adults with arthritis and thus might reduce these geographic disparities.

## Introduction

Doctor-diagnosed arthritis is a common chronic condition that affected an estimated 23% (54 million) of adults in the United States during 2013–2015 (*1*). Prevalence varies across states (*2*), counties (*2*), urban and rural communities (*3*), and census tracts within the largest U.S. cities (https://www.cdc.gov/500cities). The condition limits activities of 24 million adults (*1*), is associated with severe joint pain among 15 million adults (*4*), and is projected to affect 78.4 million adults by 2040 (*5*). In 2013, total national medical care expenditures and earnings losses attributable to arthritis were $303.5 billion (*6*). Public health measures focus on increasing physical activity, increasing self-management education, increasing health care provider counseling for physical activity or exercise, and encouraging walking.

 Since 2003, CDC has conducted surveillance for arthritis using Behavioral Risk Factor Surveillance System (BRFSS) data (*7*). The findings in this report can be used by public health professionals to better understand geographic variability in prevalence, health-related characteristics, and management of arthritis between states and territories. Public health professionals can also target evidence-based nonpharmaceutical interventions, such as arthritis self-management education and physical activity, to help decrease the impact of arthritis and perhaps reduce geographic disparities in arthritis health-related characteristics and management.

## Methods

To characterize self-reported doctor-diagnosed arthritis in the United States, Guam, and Puerto Rico, CDC analyzed data from the 2015 BRFSS (Box). First, selected area-level prevalences were estimated, including prevalences of arthritis among adults aged ≥18 years with selected comorbid conditions (i.e., coronary heart disease, diabetes, and obesity). Percentages of health-related characteristics among adults with arthritis (i.e., general health, leisure-time physical activity, activity limitations, and pain) also were estimated. Second, for 13 states with available data, self-management measures for adults with arthritis were examined. Third, to examine possible clustering of arthritis health-related characteristics linear trends were tested between increasing prevalence of arthritis and increasing percentages of negative health-related characteristics among adults with arthritis at the state level. Detailed arthritis surveillance estimates for states, the District of Columbia, Guam, and Puerto Rico are available online for 2011, 2013, and 2015 (https://www.cdc.gov/arthritis/data_statistics/state-data-list-current.htm), but are unpublished elsewhere.

BOX **Understanding geographic variations in arthritis prevalence, health-related characteristics, and management**

**Aim 1: Estimate prevalence among adults**

Prevalence of arthritis among adults with comorbid conditions (coronary heart disease, diabetes, and obesity)
Prevalence of obesity among adults with arthritis
Prevalence of leisure-time walking among adults with arthritis
Prevalence of physical inactivity among adults with arthritis
Prevalence of two types of negative health-related characteristics among adults with arthritis: general health (physically and mentally unhealthy days) and arthritis-attributable impact (activity limitations, severe joint pain, and social participation restriction)

**Aim 2: Estimate management among adults with arthritis**

Prevalence of participation in arthritis self-management education course
Prevalence of health care provider counseling for weight loss
Prevalence of health care provider counseling for physical activity or exercise

**Aim 3: Examine possible state-specific clustering of health-related characteristics among adults with arthritis**

### Data Source and Measurements

BRFSS is an annual, random-digit–dialed landline and cellular telephone survey of the noninstitutionalized U.S. adult population aged ≥18 years. Self-reported data are collected from the 50 states, the District of Columbia, Guam, and Puerto Rico. In 2015, a total of 441,456 interviews were completed and analyzed for this report. Response rates ranged from 33.9% to 61.1% (median: 47.2%). The response rate was the number of respondents who completed the survey as a proportion of all eligible and likely eligible persons. Response rates for BRFSS were calculated using standards set by American Association for Public Opinion Research response rate formula no. 4. Additional information is available at https://www.cdc.gov/brfss/annual_data/2015/2015_responserates.html.

Respondents were classified as having doctor-diagnosed arthritis (hereafter referred to as arthritis) if they answered yes to the question “Has a doctor, nurse, or other health professional ever told you that you have some form of arthritis, rheumatoid arthritis, gout, lupus, or fibromyalgia?” Three comorbid conditions were examined: obesity, diabetes, and coronary heart disease. Body mass index (BMI) was computed from self-reported height and weight. Obesity was categorized as BMI ≥30 kg/m^2^. Doctor-diagnosed diabetes (hereafter referred to as diabetes) was defined as a yes response to the question “Has a doctor, nurse, or other health professional ever told you that you have diabetes?” Those with prediabetes or borderline diabetes and women who had diabetes only during pregnancy were classified as not having diabetes. Doctor-diagnosed coronary heart disease (hereafter referred to as coronary heart disease) was defined as a yes response to either of the following two questions: 1) “Has a doctor, nurse, or other health professional ever told you that you had a heart attack, also called a myocardial infarction?” or 2) “Has a doctor, nurse, or other health professional ever told you that you had angina or coronary heart disease?”

#### Prevalence of Arthritis

The prevalence of arthritis was estimated among all adults. Prevalence was estimated separately for adults with comorbid conditions (i.e., obesity, coronary heart disease, and diabetes).

#### Health-Related Characteristics

**General Health.** Two measures of health-related quality of life were examined. For physically and mentally unhealthy days, respondents reported the number of days during the past 30 days that their physical or mental health, or both, was not good. For each measure, a standard predetermined cutoff point of ≥14 days during the past 30 days was used to identify respondents with poor physical or mental health, respectively (*8*).

**Leisure-Time Physical Activity and Obesity.** Among adults with arthritis, the prevalences of obesity, leisure-time physical inactivity, and leisure-time walking were estimated. Leisure-time physical inactivity was defined as a no response to the question “During the past month, other than your regular job, did you participate in any physical activities or exercises such as running, calisthenics, golf, gardening, or walking for exercise?” Among those who answered yes, leisure-time walking was ascertained via two questions: 1) “What type of physical activity or exercise did you spend the most time doing during the past month?” and 2) “What other type of physical activity gave you the next most exercise during the past month?” For the leisure-time walking measure, the numerator was adults with arthritis who listed walking as one of their top two activities and the denominator included both active and inactive adults with arthritis.

**Activity Limitations.** Among adults with arthritis, arthritis-attributable activity limitations were identified by a yes response to the question “Are you now limited in any way in any of your usual activities because of arthritis or joint symptoms?” Arthritis-attributable social participation restriction was defined as a response of a lot to the question “During the past 30 days, to what extent has your arthritis or joint symptoms interfered with your normal social activities, such as going shopping, to the movies, or to religious or social gatherings?”

**Pain.** Arthritis-attributable severe joint pain was defined according to an a priori criterion (*9*) as a pain level of 7–10 on a scale of 0–10 where 0 is no pain and 10 is pain or aching as bad as it can be for the question “Please think about the past 30 days, keeping in mind all of your joint pain or aching and whether or not you have taken medication. During the past 30 days, how bad was your joint pain on average?”

#### Arthritis Management

In 2015, a total of 13 states (California, Kansas, Kentucky, Michigan, Minnesota, Missouri, Montana, New York, Oregon, Pennsylvania, Rhode Island, South Carolina, and Utah) administered the BRFSS arthritis management module to respondents with arthritis and ascertained participation in self-management education courses and receipt of health care provider counseling. Among adults with arthritis, attendance at a self-management education course was defined as a yes response to the question “Have you ever taken an educational course or class to teach you how to manage problems related to your arthritis or joint symptoms?” Among those who were overweight (BMI 25 to <30 kg/m^2^) or obese (BMI ≥30 kg/m^2^), health care provider counseling for weight loss was defined as a yes response to the question “Has a doctor or other health professional ever suggested losing weight to help your arthritis or joint symptoms?” Health care provider counseling for physical activity or exercise was defined as a yes response to the question “Has a doctor or other health professional ever suggested physical activity or exercise to help your arthritis or joint symptoms?”

### Analyses

#### Direct Estimates

All directly estimated analyses included adjustment for the complex survey design; sampling weights accounted for nonresponse, noncoverage, and cellular-telephone–only households and were derived from an iterative proportional weighting (raking) procedure (https://www.cdc.gov/brfss/annual_data/2015/pdf/weighting_the-data_webpage_content.pdf). Estimates were age standardized to the 2000 U.S. projected population using three age groups (18–44, 45–64, and ≥65 years) (*10*). Weighted unadjusted and age-standardized prevalences with 95% confidence intervals were estimated for arthritis and arthritis-related characteristics. For each characteristic, the median and range were calculated using prevalence estimates for the 50 states and the District of Columbia (not including Guam and Puerto Rico). The unadjusted prevalence is an estimate of the actual prevalence of a characteristic in a specific area. Age-standardized prevalence estimates are provided to permit comparisons across states. Prevalence estimates of arthritis and percentages of selected characteristics among adults with arthritis that had a relative standard error (RSE) ≥30% or unweighted sample size of <50 did not meet the minimum criteria for precision and were suppressed.

#### Indirect (Modeled) County-Level Arthritis Prevalence Estimates

Prevalence of arthritis at the county level was estimated with a multilevel regression model and poststratification approach (*11*) for counties (N = 3,142) in all 50 states and the District of Columbia. The multilevel regression model included individual-level data on age group (13 categories), sex, and race/ethnicity from the 2015 BRFSS; county-level poverty data (percentage below 150% of the federal poverty level) from the American Community Survey 5-year estimates (2011–2015) (*12*); and random effects at county and state levels. Parameter estimates from the models were applied to Census Vintage 2015 county population estimates to generate county-level estimates of arthritis prevalence. These modeled prevalence estimates were reported in quartiles for the 3,142 counties. High internal validity was established by comparing modeled county-level estimates of arthritis with actual unweighted BRFSS survey estimates in 1,531 counties with ≥50 respondents and RSE <30% (Pearson correlation coefficient: 0.78; p<0.001) and with weighted BRFSS estimates in 205 counties with ≥500 respondents (Pearson correlation coefficient: 0.94; p<0.001).

#### State-Specific Clustering of Health-Related Characteristics

States and the District of Columbia were divided into quartiles (lowest to highest) according to age-adjusted state-level prevalence of arthritis in 2015. Age-standardized percentages of seven negative health-related characteristics among adults with arthritis (i.e., arthritis-attributable activity limitations, arthritis-attributable severe joint pain, and arthritis-attributable social participation restriction; ≥14 physically unhealthy days; ≥14 mentally unhealthy days; obesity; and leisure-time physical inactivity) and leisure-time walking were calculated for respondents by quartile of arthritis prevalence. A test of trend using orthogonal polynomial contrasts (by partitioning the sums of squares) was performed to determine whether the age-standardized prevalence of negative health-related characteristics increased and leisure-time walking decreased among adults with arthritis living in states with greater age-standardized prevalence of arthritis. To improve data fit and accommodate nonlinear trends, the test for trend included a quadratic term. For each health-related characteristic, a statistically significant trend in age-standardized percentage across arthritis quartiles was determined at the Bonferroni-corrected alpha level of 0.006 (α = 0.05/8) to adjust for testing multiple characteristics.

## Results

### Arthritis Prevalence

**FIGURE 1:**
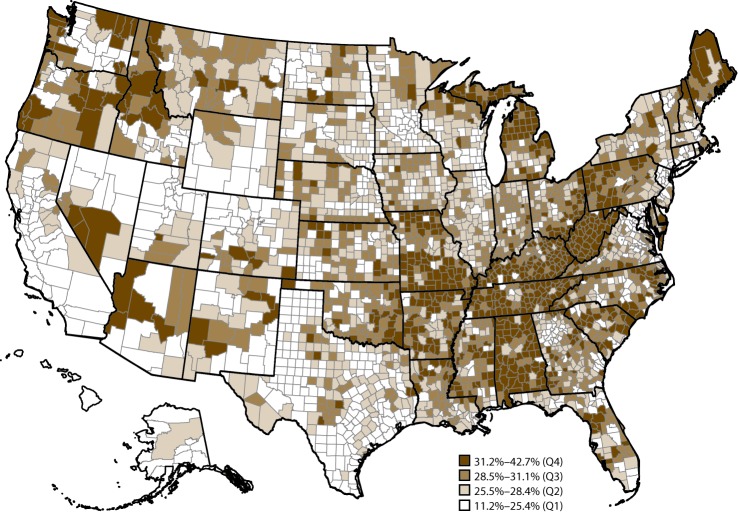
Model-based prevalence* of arthritis^†^ among adults aged ≥18 years, by county — Behavioral Risk Factor Surveillance System, United States, 2015 **Abbreviation:** Q = quartile. * Prevalence of arthritis at the county level was estimated with a multilevel regression model and poststratification approach for counties (N = 3,142) in all 50 states and the District of Columbia. Prevalence was based on the Behavioral Risk Factor Surveillance System definition of arthritis. ^†^ Doctor-diagnosed arthritis was defined as a yes response to the question “Has a doctor, nurse, or other health professional ever told you that you have some form of arthritis, rheumatoid arthritis, gout, lupus, or fibromyalgia?”

In 2015, for the 50 states and the District of Columbia, age-standardized median prevalence of arthritis was 23.0% (range: 17.2% in Hawaii to 33.6% in West Virginia) (Table 1). The model-based prevalence estimates of arthritis across the 3,142 U.S. counties in 50 states and the District of Columbia ranged from 11.2% to 42.7% (Figure 1). At the county level, counties in Appalachia and along the lower Mississippi River tended to have higher predicted prevalences of arthritis. The majority of counties in Alabama, Arkansas, Kentucky, Michigan, Missouri, Tennessee, and West Virginia were in the highest quartile (31.2%–42.7%).

**TABLE 1:** Prevalence of arthritis,* by area — Behavioral Risk Factor Surveillance System, United States, 2015

Area	No. of respondents	No. of respondents with arthritis	Weighted population with arthritis (rounded to 1,000s)	Unadjusted % (95% CI)	Age-standardized %^†^ (95% CI)
Alabama	7,950	3,307	1,248,000	33.3 (31.9–34.6)	30.4 (29.2–31.7)
Alaska	3,657	1,028	117,000	21.2 (19.3–23.2)	21.5 (19.7–23.3)
Arizona	7,946	2,663	1,222,000	23.6 (22.5–24.8)	21.8 (20.7–22.9)
Arkansas	5,256	2,228	672,000	29.7 (27.8–31.7)	27.1 (25.4–28.9)
California	12,601	2,803	5,719,000	19.1 (18.3–20.0)	18.3 (17.6–19.1)
Colorado	13,537	4,136	949,000	22.7 (21.8–23.7)	21.8 (20.9–22.7)
Connecticut	11,899	3,962	690,000	24.5 (23.5–25.5)	21.6 (20.8–22.5)
Delaware	4,070	1,471	207,000	28.1 (26.3–29.9)	24.6 (23.1–26.2)
District of Columbia	3,994	1,316	101,000	18.5 (16.7–20.4)	19.9 (18.3–21.7)
Florida	9,739	3,454	4,154,000	25.9 (24.8–27.0)	21.5 (20.6–22.5)
Georgia	4,678	1,660	1,890,000	24.6 (23.1–26.1)	23.6 (22.3–24.9)
Hawaii	7,163	1,757	211,000	18.9 (17.8–20.1)	17.2 (16.2–18.3)
Idaho	5,802	2,031	309,000	25.3 (23.8–26.8)	23.2 (22.0–24.5)
Illinois	5,289	1,671	2,308,000	23.3 (22.0–24.7)	21.6 (20.4–22.7)
Indiana	6,067	2,273	1,390,000	27.6 (26.1–29.1)	25.4 (24.1–26.7)
Iowa	6,227	2,145	619,000	25.9 (24.6–27.2)	23.2 (22.1–24.4)
Kansas	23,236	7,320	536,000	24.5 (23.9–25.2)	22.7 (22.2–23.3)
Kentucky	8,806	3,565	1,087,000	32.0 (30.5–33.5)	29.3 (27.9–30.8)
Louisiana	4,716	1,748	989,000	27.9 (26.4–29.5)	26.2 (24.8–27.7)
Maine	9,063	3,459	332,000	31.0 (29.7–32.3)	26.4 (25.2–27.6)
Maryland	12,598	4,631	1,096,000	23.5 (22.2–24.9)	21.5 (20.4–22.8)
Massachusetts	9,294	2,842	1,300,000	24.1 (23.0–25.3)	22.0 (21.0–23.0)
Michigan	8,935	3,224	2,305,000	30.0 (28.9–31.1)	27.0 (26.0–28.0)
Minnesota	16,761	4,666	907,000	21.6 (20.9–22.3)	19.7 (19.1–20.4)
Mississippi	6,035	2,431	647,000	28.6 (27.1–30.1)	26.6 (25.3–28.0)
Missouri	7,307	2,808	1,372,000	29.3 (27.9–30.8)	26.8 (25.5–28.2)
Montana	6,051	2,123	216,000	26.8 (25.4–28.3)	23.9 (22.5–25.4)
Nebraska	17,561	5,522	334,000	23.4 (22.6–24.3)	21.5 (20.7–22.3)
Nevada	2,926	918	477,000	21.5 (19.5–23.8)	20.1 (18.2–22.2)
New Hampshire	7,022	2,588	282,000	26.6 (25.3–27.9)	23.0 (21.9–24.2)
New Jersey	11,465	3,442	1,590,000	22.9 (21.8–24.1)	20.5 (19.5–21.5)
New Mexico	6,734	2,248	386,000	24.5 (23.1–25.9)	22.2 (21.0–23.5)
New York	12,357	3,921	3,629,000	23.4 (22.5–24.3)	21.5 (20.6–22.3)
North Carolina	6,698	2,144	2,089,000	26.9 (25.7–28.2)	24.9 (23.8–26.0)
North Dakota	4,972	1,585	134,000	22.9 (21.5–24.3)	21.6 (20.4–22.9)
Ohio	11,929	4,730	2,547,000	28.4 (27.2–29.7)	25.3 (24.2–26.4)
Oklahoma	6,943	2,692	813,000	27.7 (26.3–29.1)	25.7 (24.5–27.0)
Oregon	5,359	1,828	838,000	26.8 (25.4–28.2)	24.5 (23.2–25.8)
Pennsylvania	5,740	2,059	2,937,000	29.2 (27.8–30.7)	25.7 (24.4–27.0)
Rhode Island	6,206	2,244	226,000	26.9 (25.5–28.4)	24.2 (22.9–25.6)
South Carolina	11,607	4,405	1,105,000	29.1 (28.1–30.2)	26.3 (25.3–27.2)
South Dakota	7,221	2,389	158,000	24.3 (22.8–25.9)	21.8 (20.5–23.2)
Tennessee	5,979	2,466	1,630,000	32.0 (30.3–33.7)	29.4 (27.9–31.1)
Texas	14,697	4,522	4,055,000	20.0 (19.0–21.1)	19.8 (18.9–20.8)
Utah	11,401	2,929	407,000	19.6 (18.8–20.4)	20.8 (20.1–21.6)
Vermont	6,489	2,089	136,000	27.0 (25.7–28.3)	23.4 (22.3–24.6)
Virginia	8,646	2,684	1,513,000	23.2 (22.1–24.3)	21.6 (20.6–22.6)
Washington	16,116	5,481	1,346,000	24.5 (23.6–25.3)	22.6 (21.9–23.4)
West Virginia	5,957	2,537	557,000	38.0 (36.6–39.4)	33.6 (32.3–34.9)
Wisconsin	6,188	1,984	1,104,000	24.7 (23.3–26.2)	22.1 (20.8–23.5)
Wyoming	5,492	2,021	116,000	25.9 (24.2–27.5)	24.1 (22.6–25.8)
*Median (Range)^§^*				*25.3 (18.5–38.0)*	*23.0 (17.2–33.6)*
Guam	1,669	270	17,000	15.8 (13.5–18.4)	17.9 (15.5–20.6)
Puerto Rico	5,405	1,616	635,000	22.8 (21.5–24.0)	20.6 (19.5–21.7)

**Abbreviation:** CI = confidence interval.

* Doctor-diagnosed arthritis was defined as a yes response to the question “Has a doctor, nurse, or other health professional ever told you that you have some form of arthritis, rheumatoid arthritis, gout, lupus, or fibromyalgia?”

^†^ Age standardized to the 2000 U.S. projected population, using three age groups: 18–44, 45–64, and ≥65 years.

^§^ Median and range were calculated from estimates for the 50 states and the District of Columbia.

#### Arthritis Among Adults With Comorbid Conditions

For the 50 states and District of Columbia, the median age-standardized prevalence of arthritis among adults with obesity was 30.9% (range: 24.6% in Texas to 41.2% in West Virginia) (Table 2). The median age-standardized prevalence of arthritis among adults with coronary heart disease was 44.5% (range: 25.6% in the District of Columbia to 72.6% in Iowa) (Table 3). The median age-standardized prevalence of arthritis among adults with diabetes was 37.3% (range: 27.1% in California to 53.7% in Maine) (Table 4).

**TABLE 2:** Unadjusted* and age-standardized^†^ prevalences of arthritis^§^ among adults ≥18 years with obesity,^¶^ by area — Behavioral Risk Factor Surveillance System, United States, 2015

Area	No. of respondents with obesity	Weighted population with arthritis and obesity (rounded to 1,000s)	Unadjusted % (95% CI)	Age-standardized % (95% CI)
Alabama	2,626	512,000	41.4 (38.9–43.9)	37.9 (35.7–40.2)
Alaska	1,059	46,000	29.9 (26.1–34.0)	28.5 (24.9–32.4)
Arizona	1,892	398,000	30.0 (27.4–32.7)	27.5 (25.3–29.7)
Arkansas	1,593	275,000	37.7 (33.9–41.6)	35.0 (31.5–38.7)
California	2,808	1,749,000	26.4 (24.4–28.6)	24.8 (23.1–26.6)
Colorado	2,666	250,000	32.8 (30.3–35.4)	29.4 (27.2–31.7)
Connecticut	2,817	228,000	35.0 (32.7–37.4)	29.9 (27.8–32.0)
Delaware	1,145	70,000	35.6 (31.9–39.3)	30.2 (27.3–33.4)
District of Columbia	899	41,000	36.8 (31.2–42.8)	31.6 (26.6–37.0)
Florida	2,296	1,381,000	34.9 (32.4–37.6)	28.3 (26.1–30.6)
Georgia	1,340	759,000	35.4 (32.3–38.8)	31.3 (28.7–34.0)
Hawaii	1,509	67,000	27.6 (24.8–30.6)	26.8 (24.0–29.8)
Idaho	1,596	105,000	32.0 (29.0–35.1)	29.0 (26.4–31.8)
Illinois	1,547	946,000	32.4 (29.7–35.2)	28.4 (26.1–30.9)
Indiana	1,804	537,000	36.2 (33.2–39.4)	32.4 (29.7–35.3)
Iowa	1,805	241,000	34.4 (31.8–37.2)	30.5 (28.1–33.0)
Kansas	6,318	194,000	33.6 (32.2–34.9)	30.1 (28.9–31.4)
Kentucky	2,871	457,000	41.7 (38.8–44.7)	38.0 (35.2–41.0)
Louisiana	1,570	419,000	35.6 (32.6–38.7)	33.3 (30.6–36.1)
Maine	2,567	122,000	40.3 (37.8–43.0)	34.9 (32.3–37.7)
Maryland	3,437	448,000	36.9 (33.7–40.1)	32.5 (29.5–35.7)
Massachusetts	2,061	422,000	37.2 (34.4–40.2)	31.5 (28.9–34.2)
Michigan	2,582	875,000	39.3 (37.0–41.6)	34.8 (32.7–36.9)
Minnesota	4,213	321,000	32.0 (30.3–33.7)	27.7 (26.0–29.5)
Mississippi	1,964	260,000	34.4 (31.6–37.4)	32.8 (30.4–35.4)
Missouri	2,219	518,000	37.2 (34.5–40.0)	33.7 (31.1–36.3)
Montana	1,430	62,000	35.7 (32.3–39.1)	30.4 (27.2–33.8)
Nebraska	5,371	134,000	32.3 (30.5–34.2)	28.4 (26.7–30.2)
Nevada	700	155,000	28.6 (23.9–33.8)	26.4 (21.9–31.6)
New Hampshire	1,717	94,000	37.0 (34.1–40.0)	31.4 (28.7–34.3)
New Jersey	2,778	539,000	34.3 (31.6–37.0)	29.2 (26.9–31.7)
New Mexico	1,728	127,000	30.3 (27.3–33.4)	29.3 (26.6–32.2)
New York	2,933	1,221,000	34.6 (32.4–36.9)	30.9 (28.8–33.2)
North Carolina	1,808	780,000	37.2 (34.6–39.9)	33.5 (31.1–36.0)
North Dakota	1,477	51,000	30.7 (28.0–33.6)	27.2 (24.8–29.6)
Ohio	3,420	947,000	38.2 (35.7–40.8)	32.6 (30.3–35.1)
Oklahoma	2,126	329,000	35.5 (32.9–38.2)	33.0 (30.6–35.4)
Oregon	1401	302,000	35.3 (32.3–38.4)	32.0 (29.2–34.9)
Pennsylvania	5,740	1,114,000	39.6 (36.6–42.7)	33.7 (30.9–36.6)
Rhode Island	6,206	74,000	36.9 (33.6–40.3)	31.8 (28.7–35.0)
South Carolina	11,607	437,000	38.5 (36.3–40.7)	34.9 (32.9–36.9)
South Dakota	7,221	60,000	31.7 (28.6–35.0)	28.3 (25.6–31.1)
Tennessee	5,979	640,000	40.7 (37.4–44.1)	37.6 (34.5–40.9)
Texas	14,697	1,518,000	26.2 (24.0–28.4)	24.6 (22.9–26.4)
Utah	11,401	137,000	29.3 (27.3–31.4)	28.0 (26.2–29.9)
Vermont	6,489	43,000	37.1 (34.1–40.1)	31.2 (28.4–34.2)
Virginia	8,646	584,000	33.0 (30.5–35.5)	29.6 (27.2–32.0)
Washington	16,116	454,000	34.5 (32.6–36.5)	29.8 (28.1–31.5)
West Virginia	5,957	223,000	46.6 (44.1–49.2)	41.2 (38.7–43.7)
Wisconsin	6,188	417,000	32.6 (29.8–35.6)	28.2 (25.6–31.1)
Wyoming	5,492	41,000	34.1 (30.6–37.7)	31.2 (28.1–34.5)
Median (Range)**			*35.0 (26.2–46.6)*	*30.9 (24.6–41.2)*
Guam	1,669	7,000	20.3 (15.9–25.5)	23.4 (19.0–28.5)
Puerto Rico	5,405	215,000	27.5 (25.0–30.2)	25.7 (23.4–28.2)

**Abbreviation:** CI = confidence interval.

* The numerator was the estimated number of adults with arthritis and obesity (body mass index ≥30). The denominator was the estimated number of adults with obesity.

^†^ Age standardized to the 2000 U.S. projected population, using three age groups: 18–44, 45–64, and ≥65 years.

^§^ Doctor-diagnosed arthritis was defined as a yes response to the question “Has a doctor, nurse, or other health professional ever told you that you have some form of arthritis, rheumatoid arthritis, gout, lupus, or fibromyalgia?”

^¶^ Body mass index ≥30; calculated from self-reported height and weight.

** Median and range were calculated from estimates for the 50 states and the District of Columbia.

**TABLE 3:** Unadjusted* and age-standardized^†^ prevalences of arthritis^§^ among adults aged ≥18 years with coronary heart disease,^¶^ by area — Behavioral Risk Factor Surveillance System, United States, 2015

Area	No. of respondents with coronary heart disease	Weighted population with arthritis and coronary heart disease (rounded to 1,000s)	Unadjusted % (95% CI)	Age-standardized %* (95% CI)
Alabama	874	196,000	64.3 (59.7–68.7)	57.0 (47.0–66.6)
Alaska	223	12,000	49.1 (38.0–60.2)	31.3 (21.8–42.6)
Arizona	732	164,000	51.6 (46.1–57.1)	36.9 (27.8–47.0)
Arkansas	714	122,000	64.6 (58.5–70.3)	57.8 (41.8–72.4)
California	701	708,000	49.0 (43.8–54.2)	38.1 (28.7–48.5)
Colorado	857	99,000	53.4 (48.3–58.4)	48.2 (35.9–60.7)
Connecticut	945	80,000	52.1 (47.7–56.5)	41.2 (30.8–52.4)
Delaware	385	30,000	57.5 (49.5–65.1)	44.4 (27.2–63.0)
District of Columbia	286	11,000	45.9 (34.8–57.3)	25.6 (20.0–32.3)
Florida	1,031	667,000	56.2 (51.9–60.5)	43.0 (31.8–54.9)
Georgia	454	301,000	59.0 (52.6–65.0)	48.3 (34.6–62.3)
Hawaii	409	22,000	43.5 (36.7–50.5)	39.1 (26.9–52.8)
Idaho	469	37,000	53.8 (46.4–61.0)	36.7 (27.2–47.5)
Illinois	457	317,000	53.0 (47.0–58.9)	39.1 (26.0–54.1)
Indiana	672	239,000	60.9 (55.4–66.2)	52.2 (39.6–64.5)
Iowa	516	86,000	59.9 (54.6–65.0)	72.6 (60.6–82.1)
Kansas	1,894	72,000	56.5 (53.8–59.1)	43.5 (36.8–50.3)
Kentucky	1,055	187,000	59.3 (54.3–64.1)	48.4 (38.9–57.9)
Louisiana	527	173,000	61.1 (55.4–66.5)	55.8 (43.2–67.7)
Maine	824	51,000	60.8 (56.1–65.3)	52.6 (40.0–65.0)
Maryland	1,126	140,000	52.3 (46.5–58.0)	38.9 (27.5–51.7)
Massachusetts	656	181,000	56.8 (51.0–62.4)	48.1 (35.1–61.5)
Michigan	788	344,000	62.1 (57.9–66.2)	45.7 (35.2–56.6)
Minnesota	1,131	110,000	50.6 (47.0–54.1)	35.8 (27.9–44.7)
Mississippi	659	110,000	58.2 (52.3–63.9)	43.5 (34.0–53.4)
Missouri	780	204,000	58.3 (53.3–63.1)	38.2 (29.2–48.1)
Montana	502	26,000	57.9 (51.6–63.9)	44.0 (27.3–62.2)
Nebraska	1,447	43,000	51.8 (47.9–55.7)	35.8 (29.5–42.7)
Nevada	247	86,000	63.0 (52.9–72.0)	41.6 (27.5–57.3)
New Hampshire	614	33,000	55.2 (49.9–60.3)	43.4 (27.6–60.7)
New Jersey	848	215,000	55.9 (50.6–61.0)	50.4 (36.5–64.2)
New Mexico	572	50,000	56.0 (49.8–62.0)	30.5 (25.8–35.7)
New York	959	510,000	57.3 (53.0–61.6)	56.1 (44.4–67.3)
North Carolina	580	313,000	57.9 (52.8–62.8)	44.5 (33.4–56.2)
North Dakota	414	18,000	54.1 (47.9–60.1)	55.2 (35.8–73.2)
Ohio	1,295	382,000	61.0 (56.2–65.6)	45.3 (32.4–58.8)
Oklahoma	838	161,000	65.3 (60.2–70.2)	47.9 (36.4–59.6)
Oregon	398	97,000	56.4 (50.1–62.6)	46.4 (30.9–62.7)
Pennsylvania	485	412,000	58.4 (52.4–64.2)	49.9 (33.5–66.3)
Rhode Island	534	30,000	59.8 (53.7–65.6)	60.3 (44.3–74.3)
South Carolina	1,134	162,000	62.0 (58.0–65.8)	51.0 (41.3–60.7)
South Dakota	715	28,000	61.1 (54.9–66.9)	50.8 (36.6–64.9)
Tennessee	703	271,000	63.2 (58.1–68.0)	55.1 (42.7–66.9)
Texas	1,362	606,000	49.9 (44.3–55.5)	36.8 (28.7–45.8)
Utah	618	44,000	53.4 (48.3–58.5)	40.2 (30.6–50.6)
Vermont	477	19,000	57.8 (52.0–63.3)	43.8 (30.9–57.6)
Virginia	637	182,000	51.5 (46.4–56.5)	36.5 (28.9–44.8)
Washington	1,310	180,000	57.4 (53.5–61.3)	47.1 (38.7–55.8)
West Virginia	755	102,000	63.8 (59.7–67.7)	53.7 (44.1–63.1)
Wisconsin	494	134,000	48.9 (42.6–55.2)	35.9 (25.7–47.7)
Wyoming	564	16,000	58.6 (52.0–64.9)	58.6 (39.4–75.5)
Median (Range)**			*57.4 (43.5–65.3)*	*44.5 (25.6–72.6)*
Guam	88	3,000	49.1 (33.5–64.9)	42.2 (22.3–65.0)
Puerto Rico	586	126,000	49.2 (44.3–54.2)	37.3 (30.2–44.9)

**Abbreviation:** CI = confidence interval.

* The numerator was the estimated number of adults with arthritis and coronary heart disease. The denominator was the estimated number of adults with coronary heart disease.

^†^ Age standardized to the 2000 U.S. projected population, using three age groups: 18–44, 45–64, and ≥65 years.

^§^ Doctor-diagnosed arthritis was defined as a yes response to the question “Has a doctor, nurse, or other health professional ever told you that you have some form of arthritis, rheumatoid arthritis, gout, lupus, or fibromyalgia?”

^¶^ Doctor-diagnosed coronary heart disease was defined as a yes response to either of the following two questions: “Has a doctor, nurse, or other health professional ever told you that you had a heart attack, also called a myocardial infarction?” or “Has a doctor, nurse, or other health professional ever told you that you had angina or coronary heart disease?” Those who answered yes to either question were classified as having coronary heart disease. Those who answered no to both questions were classified as not having coronary heart disease.

** Median and range were calculated from estimates for the 50 states and the District of Columbia.

**TABLE 4:** Unadjusted* and age-standardized^†^ prevalences of arthritis^§^ among adults aged ≥18 years with diabetes,^¶^ by area — Behavioral Risk Factor Surveillance System, United States, 2015

Area	No. of respondents with diabetes	Weighted population with arthritis and diabetes (rounded to 1,000s)	Unadjusted % (95% CI)	Age-standardized %* (95% CI)
Alabama	1,355	283,000	56.4 (52.7–60.0)	43.8 (38.0–49.7)
Alaska	346	19,000	44.4 (35.3–53.8)	46.0 (29.6–63.4)
Arizona	1,095	238,000	45.7 (41.6–49.9)	32.6 (27.0–38.8)
Arkansas	951	167,000	58.9 (53.7–64.0)	48.6 (38.4–58.9)
California	1,283	1,088,000	36.4 (32.8–40.2)	27.1 (23.1–31.5)
Colorado	1,216	132,000	47.0 (42.9–51.1)	37.3 (30.3–44.9)
Connecticut	1,379	117,000	45.5 (41.8–49.2)	30.1 (25.0–35.6)
Delaware	606	42,000	50.0 (44.5–55.4)	35.4 (26.8–44.9)
District of Columbia	544	22,000	47.0 (39.2–55.0)	28.2 (22.8–34.2)
Florida	1,394	958,000	53.0 (49.2–56.7)	35.6 (28.9–43.0)
Georgia	752	453,000	52.2 (47.4–56.9)	40.0 (30.8–49.9)
Hawaii	722	37,000	38.7 (34.0–43.7)	32.0 (24.9–40.0)
Idaho	678	46,000	46.8 (41.7–52.0)	37.0 (28.6–46.2)
Illinois	680	452,000	46.1 (41.3–50.9)	34.2 (25.7–44.0)
Indiana	885	297,000	51.5 (46.6–56.3)	37.6 (30.9–44.9)
Iowa	753	110,000	52.1 (47.4–56.7)	32.2 (26.3–38.7)
Kansas	2,863	106,000	50.4 (48.2–52.6)	37.1 (33.8–40.6)
Kentucky	1,457	253,000	55.9 (51.5–60.3)	45.4 (37.9–53.0)
Louisiana	793	241,000	53.7 (49.1–58.4)	41.5 (33.7–49.7)
Maine	1,083	62,000	58.2 (54.0–62.2)	53.7 (43.8–63.3)
Maryland	1,854	207,000	43.5 (39.1–48.1)	34.8 (27.2–43.3)
Massachusetts	983	230,000	48.4 (43.8–53.0)	37.0 (30.3–44.2)
Michigan	1,087	438,000	53.2 (49.5–56.9)	40.6 (34.9–46.5)
Minnesota	1,637	150,000	47.8 (44.8–50.9)	36.9 (30.6–43.6)
Mississippi	1,151	172,000	51.7 (47.5–55.8)	39.7 (33.6–46.0)
Missouri	1,154	297,000	55.5 (51.3–59.6)	53.7 (44.9–62.2)
Montana	652	34,000	53.9 (48.2–59.5)	46.4 (34.2–59.1)
Nebraska	2,046	60,000	48.0 (44.7–51.3)	30.7 (26.5–35.1)
Nevada	337	89,000	41.8 (33.9–50.1)	28.9 (19.1–41.2)
New Hampshire	834	46,000	53.2 (48.6–57.8)	36.3 (28.4–45.0)
New Jersey	1,314	302,000	48.3 (44.1–52.6)	38.3 (29.4–48.0)
New Mexico	919	83,000	46.5 (41.7–51.3)	33.1 (27.2–39.5)
New York	1,469	730,000	48.1 (44.6–51.6)	38.8 (32.4–45.7)
North Carolina	855	440,000	53.4 (49.4–57.4)	40.5 (33.9–47.6)
North Dakota	565	22,000	43.8 (38.6–49.1)	27.4 (21.7–33.9)
Ohio	1,861	531,000	53.8 (50.1–57.5)	39.7 (33.0–46.9)
Oklahoma	1,091	188,000	55.0 (50.9–59.0)	44.2 (36.6–52.1)
Oregon	655	157,000	48.0 (43.1–52.9)	40.0 (31.1–49.5)
Pennsylvania	715	575,000	55.2 (50.3–60.1)	40.0 (31.0–49.7)
Rhode Island	748	39,000	51.5 (46.4–56.6)	42.9 (29.9–56.9)
South Carolina	1,837	254,000	56.9 (53.6–60.2)	47.6 (41.4–54.0)
South Dakota	862	28,000	46.9 (41.2–52.6)	31.6 (25.4–38.4)
Tennessee	998	348,000	54.3 (49.6–59.0)	42.2 (35.4–49.4)
Texas	2,269	977,000	42.4 (38.4–46.6)	27.8 (24.4–31.5)
Utah	1,018	68,000	46.7 (42.8–50.6)	33.0 (27.5–39.0)
Vermont	625	20,000	49.3 (44.4–54.3)	37.5 (30.1–45.5)
Virginia	1,129	328,000	48.8 (44.7–53.0)	34.3 (29.6–39.4)
Washington	1,782	219,000	47.8 (44.5–51.0)	35.4 (30.2–41.1)
West Virginia	962	132,000	62.4 (58.8–65.9)	52.3 (46.1–58.4)
Wisconsin	678	161,000	43.4 (38.2–48.8)	28.3 (22.9–34.4)
Wyoming	678	19,000	51.7 (46.1–57.2)	47.2 (33.6–61.3)
Median (Range)**			*49.3 (36.4–62.4)*	*37.3 (27.1–53.7)*
Guam	192	5,000	39.1 (29.7–49.4)	26.8 (19.2–36.1)
Puerto Rico	1,084	206,000	45.3 (41.8–48.9)	28.1 (23.9–32.8)

**Abbreviation:** CI = confidence interval.

* The numerator was the estimated number of adults with arthritis and diabetes. The denominator was the estimated number of adults with diabetes.

^†^ Age standardized to the 2000 U.S. projected population, using three age groups: 18–44, 45–64, and ≥65 years.

^§^ Doctor-diagnosed arthritis was defined as a yes response to the question “Has a doctor, nurse, or other health professional ever told you that you have some form of arthritis, rheumatoid arthritis, gout, lupus, or fibromyalgia?”

^¶^ Doctor-diagnosed diabetes was defined as a yes response to the question “Has a doctor, nurse, or other health professional ever told you that you have diabetes?” Those with prediabetes or borderline diabetes and women who had diabetes only during pregnancy were classified as not having diabetes.

** Median and range were calculated from estimates for the 50 states and the District of Columbia.

### Health-Related Characteristics

#### General Health

In 2015, the percentage of poor health-related quality of life among adults with arthritis varied substantially by state. The median age-standardized percentage of ≥14 physically unhealthy days during the past 30 days was 27.7% (range: 16.9% in Alaska to 37.5% in Oklahoma) (Table 5). The median age-standardized percentage of ≥14 mentally unhealthy days during the past 30 days was 22.3% (range: 14.8% in Hawaii to 31.1% Mississippi) (Table 6).

**TABLE 5:** Unadjusted and age-standardized* percentages of ≥14 physically unhealthy days^†^ during the past 30 days among adults aged ≥18 years with arthritis,^§^ by area — Behavioral Risk Factor Surveillance System, United States, 2015

Area	Weighted population with arthritis (rounded to 1,000s)	Weighted population with arthritis and ≥14 physically unhealthy days (rounded to 1,000s)	Unadjusted % (95% CI)	Age-standardized % (95% CI)
Alabama	1,248,000	365,000	30.5 (28.3–32.7)	30.6 (27.2–34.2)
Alaska	117,000	23,000	21.3 (17.9–25.2)	16.9 (13.4–21.2)
Arizona	1,222,000	354,000	29.7 (27.2–32.3)	30.1 (25.8–34.8)
Arkansas	672,000	208,000	32.3 (29.0–35.8)	35.7 (29.8–42.1)
California	5,719,000	1,383,000	24.5 (22.5–26.7)	24.6 (21.3–28.2)
Colorado	949,000	232,000	25.1 (23.1–27.3)	27.7 (23.9–31.7)
Connecticut	690,000	157,000	23.4 (21.5–25.5)	25.1 (21.3–29.2)
Delaware	207,000	48,000	23.9 (21.0–27.0)	24.8 (19.9–30.5)
District of Columbia	101,000	25,000	25.6 (21.2–30.6)	23.0 (15.3–33.1)
Florida	4,154,000	1,190,000	30.0 (27.8–32.4)	33.6 (28.8–38.9)
Georgia	1,890,000	526,000	28.7 (25.8–31.8)	25.0 (20.7–29.9)
Hawaii	211,000	45,000	21.4 (18.8–24.3)	21.0 (16.7–26.2)
Idaho	309,000	68,000	22.8 (20.3–25.4)	24.7 (20.3–29.6)
Illinois	2,308,000	595,000	26.0 (23.2–28.9)	24.8 (19.9–30.4)
Indiana	1,390,000	377,000	28.3 (25.6–31.1)	30.1 (24.9–35.8)
Iowa	619,000	131,000	21.9 (19.7–24.2)	20.4 (16.6–24.7)
Kansas	536,000	119,000	23.0 (21.8–24.2)	22.9 (20.9–25.1)
Kentucky	1,087,000	338,000	31.9 (29.4–34.5)	30.2 (26.2–34.5)
Louisiana	989,000	288,000	30.5 (27.6–33.4)	29.8 (25.3–34.7)
Maine	332,000	82,000	25.2 (23.1–27.5)	29.1 (24.9–33.7)
Maryland	1,096,000	260,000	24.7 (22.0–27.5)	28.7 (23.1–35.0)
Massachusetts	1,300,000	325,000	26.1 (23.8–28.6)	29.0 (25.0–33.3)
Michigan	2,305,000	622,000	27.5 (25.6–29.5)	29.8 (26.5–33.2)
Minnesota	907,000	218,000	24.8 (23.1–26.5)	27.1 (23.7–30.6)
Mississippi	647,000	224,000	35.6 (32.8–38.5)	35.5 (30.8–40.6)
Missouri	1,372,000	390,000	29.1 (26.7–31.7)	29.3 (24.9–34.0)
Montana	216,000	54,000	25.5 (22.8–28.4)	26.7 (21.9–32.1)
Nebraska	334,000	70,000	21.3 (19.7–23.0)	20.3 (17.5–23.4)
Nevada	477,000	126,000	27.5 (22.9–32.5)	30.6 (22.6–40.0)
New Hampshire	282,000	64,000	23.1 (20.9–25.6)	25.5 (20.9–30.8)
New Jersey	1,590,000	378,000	24.8 (22.5–27.1)	24.4 (20.9–28.3)
New Mexico	386,000	111,000	29.3 (26.7–32.1)	27.8 (23.2–33.0)
New York	3,629,000	910,000	26.5 (24.5–28.6)	30.0 (26.0–34.2)
North Carolina	2,089,000	607,000	30.2 (27.7–32.7)	27.8 (24.2–31.6)
North Dakota	134,000	28,000	21.5 (18.8–24.4)	21.7 (17.1–27.0)
Ohio	2,547,000	664,000	26.8 (24.6–29.1)	27.0 (22.8–31.6)
Oklahoma	813,000	278,000	35.2 (32.5–38.0)	37.5 (32.7–42.6)
Oregon	838,000	238,000	29.6 (26.9–32.5)	30.2 (25.8–35.0)
Pennsylvania	2,937,000	682,000	23.8 (21.5–26.4)	24.4 (20.4–28.8)
Rhode Island	226,000	61,000	28.4 (25.6–31.3)	32.7 (27.1–38.9)
South Carolina	1,105,000	332,000	31.1 (29.2–33.2)	31.3 (27.8–35.0)
South Dakota	158,000	35,000	22.4 (19.7–25.5)	20.3 (16.5–24.7)
Tennessee	1,630,000	521,000	33.0 (30.2–36.0)	31.9 (27.4–36.8)
Texas	4,055,000	1,035,000	26.7 (24.2–29.3)	25.9 (22.0–30.3)
Utah	407,000	98,000	24.8 (22.8–27.0)	25.3 (22.4–28.4)
Vermont	136,000	33,000	25.3 (22.8–28.0)	26.5 (21.9–31.6)
Virginia	1,513,000	359,000	24.3 (22.1–26.6)	24.5 (20.6–28.8)
Washington	1,346,000	332,000	25.2 (23.5–26.9)	27.7 (24.3–31.3)
West Virginia	557,000	186,000	34.1 (32.0–36.4)	33.1 (29.8–36.5)
Wisconsin	1,104,000	264,000	24.2 (21.7–27.0)	21.1 (17.6–25.0)
Wyoming	116,000	30,000	26.5 (23.3–29.9)	28.9 (23.1–35.4)
*Median (Range)^¶^*			*26.0 (21.3–35.6)*	*27.7 (16.9–37.5)*
Guam	17,000	4,000	23.8 (17.5–31.4)	21.5 (14.8–30.2)
Puerto Rico	635,000	196,000	31.0 (28.3–33.8)	30.6 (25.8–35.9)

**Abbreviation:** CI = confidence interval.

* Age standardized to the 2000 U.S. projected population, using three age groups: 18–44, 45–64, and ≥65 years.

^†^ Respondents with arthritis who answered 14–30 days to the question “Now thinking about your physical health, which includes physical illness and injury, for how many days during the past 30 days was your physical health not good?”

^§^ Doctor-diagnosed arthritis was defined as a yes response to the question “Has a doctor, nurse, or other health professional ever told you that you have some form of arthritis, rheumatoid arthritis, gout, lupus, or fibromyalgia?”

^¶^ Median and range were calculated from estimates for the 50 states and the District of Columbia.

**TABLE 6:** Unadjusted and age-standardized* percentages of ≥14 mentally unhealthy days^†^ during the past 30 days among adults aged ≥18 years with arthritis,^§^ by area — Behavioral Risk Factor Surveillance System, United States, 2015

**Area**	**Weighted population with arthritis (rounded to 1,000s)**	**Weighted population with arthritis and ≥14 mentally unhealthy days (rounded to 1,000s)**	**Unadjusted % (95% CI)**	**Age-standardized % (95% CI)**
Alabama	1,248,000	268,000	22.0 (20.0–24.1)	29.4 (25.8–33.2)
Alaska	117,000	19,000	16.6 (13.2–20.7)	18.0 (13.0–24.4)
Arizona	1,222,000	210,000	17.5 (15.5–19.7)	21.0 (17.3–25.4)
Arkansas	672,000	138,000	21.2 (18.2–24.5)	26.6 (21.2–32.7)
California	5,719,000	934,000	16.6 (14.8–18.5)	21.7 (18.4–25.4)
Colorado	949,000	145,000	15.7 (13.8–17.7)	23.1 (19.3–27.4)
Connecticut	690,000	104,000	15.3 (13.7–17.0)	21.1 (17.7–25.0)
Delaware	207,000	36,000	17.5 (15.0–20.4)	20.1 (15.4–25.7)
District of Columbia	101,000	17,000	17.5 (13.5–22.3)	20.1 (12.4–31.0)
Florida	4,154,000	772,000	19.0 (17.1–21.2)	23.9 (20.0–28.3)
Georgia	1,890,000	343,000	18.8 (16.2–21.7)	21.0 (16.1–26.9)
Hawaii	211,000	24,000	11.7 (9.7–13.9)	14.8 (10.9–19.9)
Idaho	309,000	46,000	15.3 (13.0–17.9)	22.2 (17.5–27.7)
Illinois	2,308,000	330,000	14.6 (12.4–17.0)	19.3 (15.0–24.5)
Indiana	1,390,000	241,000	17.8 (15.5–20.4)	24.4 (19.4–30.2)
Iowa	619,000	85,000	13.9 (12.0–16.1)	22.3 (17.8–27.6)
Kansas	536,000	77,000	14.6 (13.6–15.7)	20.3 (18.2–22.6)
Kentucky	1,087,000	224,000	21.1 (18.6–23.8)	26.5 (21.9–31.7)
Louisiana	989,000	199,000	20.6 (18.2–23.3)	25.6 (21.2–30.6)
Maine	332,000	57,000	17.6 (15.7–19.6)	25.0 (21.1–29.4)
Maryland	1,096,000	204,000	19.0 (16.5–21.9)	29.9 (24.2–36.2)
Massachusetts	1,300,000	238,000	18.8 (16.7–21.1)	26.3 (22.3–30.9)
Michigan	2,305,000	408,000	18.0 (16.3–19.8)	24.0 (20.9–27.4)
Minnesota	907,000	118,000	13.3 (12.0–14.7)	19.5 (16.6–22.7)
Mississippi	647,000	148,000	23.6 (21.0–26.4)	31.1 (26.3–36.3)
Missouri	1,372,000	263,000	19.6 (17.2–22.1)	25.8 (21.1–31.1)
Montana	216,000	34,000	16.0 (13.6–18.7)	21.8 (16.9–27.7)
Nebraska	334,000	43,000	13.1 (11.8–14.6)	16.7 (13.9–20.1)
Nevada	477,000	93,000	19.9 (15.4–25.3)	30.0 (21.5–40.2)
New Hampshire	282,000	45,000	16.3 (14.3–18.5)	24.1 (19.2–29.8)
New Jersey	1,590,000	249,000	16.0 (14.1–18.2)	21.3 (17.2–26.2)
New Mexico	386,000	65,000	17.2 (15.0–19.7)	21.6 (17.2–26.8)
New York	3,629,000	636,000	18.2 (16.4–20.1)	23.3 (19.9–27.0)
North Carolina	2,089,000	404,000	19.7 (17.6–22.0)	24.6 (20.8–28.8)
North Dakota	134,000	17,000	13.3 (10.7–16.4)	21.7 (16.2–28.3)
Ohio	2,547,000	470,000	18.9 (16.8–21.1)	25.6 (21.3–30.3)
Oklahoma	813,000	179,000	22.6 (20.2–25.2)	28.6 (24.1–33.5)
Oregon	838,000	167,000	20.6 (18.1–23.3)	27.4 (22.9–32.5)
Pennsylvania	2,937,000	478,000	16.6 (14.5–18.9)	21.0 (17.0–25.5)
Rhode Island	226,000	41,000	18.4 (16.0–21.2)	26.9 (21.5–33.2)
South Carolina	1,105,000	238,000	22.3 (20.5–24.2)	28.4 (24.9–32.3)
South Dakota	158,000	18,000	11.3 (9.2–13.9)	15.7 (11.6–21.1)
Tennessee	1,630,000	337,000	21.0 (18.6–23.7)	25.8 (21.3–30.8)
Texas	4,055,000	658,000	16.7 (14.6–19.1)	23.2 (18.8–28.3)
Utah	407,000	66,000	16.7 (14.9–18.6)	20.4 (17.6–23.5)
Vermont	136,000	20,000	15.1 (13.1–17.2)	20.3 (16.4–24.9)
Virginia	1,513,000	207,000	14.0 (12.2–15.9)	19.7 (16.2–23.8)
Washington	1,346,000	211,000	15.9 (14.5–17.4)	20.8 (17.9–24.1)
West Virginia	557,000	127,000	23.5 (21.5–25.5)	28.5 (25.3–32.0)
Wisconsin	1,104,000	173,000	15.8 (13.5–18.4)	22.0 (17.2–27.6)
Wyoming	116,000	18,000	15.8 (13.3–18.7)	20.0 (15.5–25.5)
*Median (Range)^¶^*			*17.5 (11.3–23.6)*	*22.3 (14.8–31.1)*
Guam	17,000	3,000	15.5 (10.9–21.5)	18.1 (11.5–27.3)
Puerto Rico	635,000	144,000	23.0 (20.5–25.6)	27.7 (22.9–33.0)

**Abbreviation:** CI = confidence interval.

* Age standardized to the 2000 U.S. projected population, using three age groups: 18–44, 45–64, and ≥65 years.

^†^ Respondents with arthritis who answered 14–30 days to the question “Now thinking about your mental health, which includes stress, depression, and problems with emotions, for how many days during the past 30 days was your mental health not good?”

^§^ Doctor-diagnosed arthritis was defined as a yes response to the question “Has a doctor, nurse, or other health professional ever told you that you have some form of arthritis, rheumatoid arthritis, gout, lupus, or fibromyalgia?”

^¶^ Median and range were calculated from estimates for the 50 states and the District of Columbia.

#### Leisure-Time Physical Activity and Obesity

**FIGURE 2:**
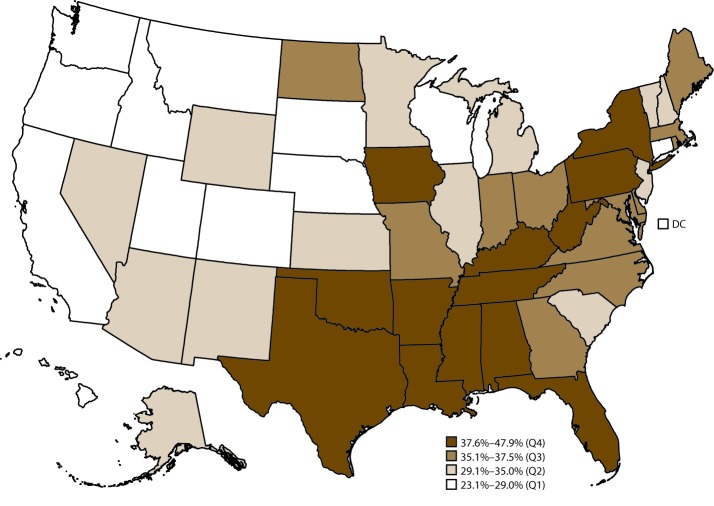
Age-standardized prevalence of physical inactivity* among adults aged ≥18 years with arthritis, by state — Behavioral Risk Factor Surveillance System, United States, 2015 **Abbreviations:** DC = District of Columbia; Q = quartile. * Physical inactivity was defined as a no response to the question “During the past month, other than your regular job, did you participate in any physical activities or exercises such as running, calisthenics, golf, gardening, or walking for exercise?”

**FIGURE 3:**
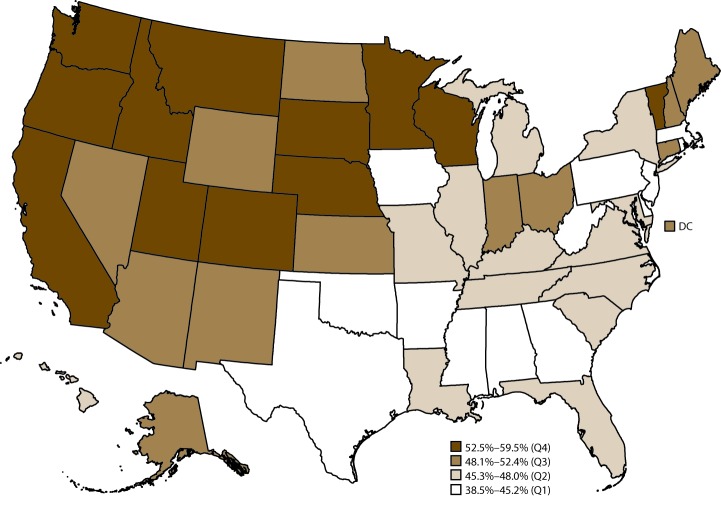
Age-standardized prevalence of walking* among adults aged ≥18 years with arthritis, by state — Behavioral Risk Factor Surveillance System, United States, 2015 **Abbreviations:** DC = District of Columbia; Q = quartile. * Respondents with arthritis who reported walking or hiking for one of two questions: 1) “What type of physical activity or exercise did you spend the most time doing during the past month?” and 2) “What other type of physical activity gave you the next most exercise during the past month?”

In 2015, for leisure-time physical inactivity, the median age-standardized percentage among adults with arthritis was 35.0% (range: 23.1% in California to 47.9% in Mississippi) (Table 7). States in the western United States (e.g., California, Idaho, Oregon, and Washington) tended to have the lowest prevalence of leisure-time physical inactivity among adults with arthritis, whereas states primarily in Appalachia and along the Ohio River and Mississippi River had the highest percentage of leisure-time physical inactivity (Figure 2). Age-standardized percentage of leisure-time physical inactivity was ≥40% in Alabama, Arkansas, Louisiana, Mississippi, Oklahoma, and Texas. For the 50 states and the District of Columbia, the median age-standardized percentage of leisure-time walking was 48.0% (range: 38.5% in West Virginia to 59.5% in Montana) (Table 8). Leisure-time walking tended to be highest in western states (e.g., California, Idaho, Oregon, and Washington) and lowest in states primarily in Appalachia and along the Ohio River and Mississippi River (e.g., Alabama, Arkansas, Mississippi, and West Virginia) (Figure 3).

**TABLE 7:** Unadjusted and age-standardized* percentages of physical inactivity^†^ among adults aged ≥18 years with arthritis,^§^ by area — Behavioral Risk Factor Surveillance System, United States, 2015

Area	Weighted population with arthritis (rounded to 1,000s)	Weighted population with arthritis and physical inactivity (rounded to 1,000s)	Unadjusted % (95% CI)	Age-standardized % (95% CI)
Alabama	1,248,000	488,000	43.7 (41.2–46.2)	40.0 (36.2–44.0)
Alaska	117,000	33,000	31.8 (27.1–36.9)	30.8 (24.1–38.5)
Arizona	1,222,000	357,000	33.7 (31.1–36.4)	31.8 (27.0–36.9)
Arkansas	672,000	280,000	47.6 (43.8–51.5)	44.3 (38.0–50.8)
California	5,719,000	1,217,000	24.7 (22.5–27.1)	23.1 (19.8–26.9)
Colorado	949,000	218,000	27.0 (24.9–29.3)	25.2 (21.8–28.9)
Connecticut	690,000	195,000	32.5 (30.4–34.7)	26.7 (23.4–30.2)
Delaware	207,000	78,000	41.8 (38.2–45.5)	37.5 (31.1–44.3)
District of Columbia	101,000	25,000	30.9 (26.1–36.1)	24.9 (16.2–36.2)
Florida	4,154,000	1,396,000	39.1 (36.6–41.7)	36.6 (31.3–42.2)
Georgia	1,890,000	650,000	38.9 (35.6–42.3)	35.9 (29.6–42.6)
Hawaii	211,000	50,000	26.0 (23.2–29.0)	29.0 (23.7–34.8)
Idaho	309,000	87,000	31.1 (28.2–34.1)	28.6 (23.9–33.8)
Illinois	2,308,000	768,000	36.4 (33.4–39.5)	33.4 (28.1–39.0)
Indiana	1,390,000	505,000	40.9 (37.8–44.0)	37.0 (31.3–43.1)
Iowa	619,000	205,000	37.5 (34.7–40.3)	38.5 (33.0–44.3)
Kansas	536,000	173,000	36.9 (35.5–38.3)	33.0 (30.6–35.5)
Kentucky	1,087,000	423,000	44.1 (41.2–47.0)	39.9 (35.4–44.7)
Louisiana	989,000	375,000	44.6 (41.3–48.0)	40.1 (34.7–45.6)
Maine	332,000	111,000	36.9 (34.5–39.2)	36.0 (31.7–40.5)
Maryland	1,096,000	355,000	38.0 (34.9–41.2)	37.1 (31.2–43.5)
Massachusetts	1,300,000	408,000	37.7 (34.9–40.5)	36.0 (31.3–41.0)
Michigan	2,305,000	747,000	35.7 (33.5–37.9)	34.0 (30.5–37.7)
Minnesota	907,000	275,000	33.1 (31.3–35.0)	30.7 (27.3–34.3)
Mississippi	647,000	297,000	50.1 (47.0–53.1)	47.9 (42.7–53.1)
Missouri	1,372,000	466,000	37.3 (34.6–40.2)	36.6 (31.6–41.9)
Montana	216,000	61,000	30.3 (27.4–33.4)	28.9 (24.0–34.4)
Nebraska	334,000	104,000	34.0 (32.1–36.0)	28.5 (25.2–32.2)
Nevada	477,000	140,000	33.0 (27.9–38.5)	31.0 (23.1–40.1)
New Hampshire	282,000	87,000	35.0 (32.3–37.7)	33.0 (27.7–38.8)
New Jersey	1,590,000	523,000	37.1 (34.4–39.8)	32.8 (28.4–37.4)
New Mexico	386,000	104,000	30.3 (27.6–33.2)	29.1 (24.1–34.7)
New York	3,629,000	1,241,000	41.1 (38.8–43.4)	39.7 (35.3–44.1)
North Carolina	2,089,000	749,000	39.9 (37.2–42.7)	37.1 (32.8–41.6)
North Dakota	134,000	45,000	37.4 (34.1–40.7)	35.1 (29.3–41.4)
Ohio	2,547,000	884,000	39.2 (36.7–41.7)	35.7 (31.3–40.5)
Oklahoma	813,000	340,000	45.9 (43.1–48.7)	41.9 (37.1–46.8)
Oregon	838,000	193,000	26.8 (24.0–29.7)	24.0 (19.9–28.7)
Pennsylvania	2,937,000	1,010,000	39.2 (36.3–42.3)	38.0 (32.9–43.4)
Rhode Island	226,000	75,000	38.5 (35.5–41.7)	36.8 (30.5–43.6)
South Carolina	1,105,000	373,000	37.6 (35.5–39.7)	35.0 (31.4–38.7)
South Dakota	158,000	43,000	29.7 (26.4–33.2)	23.4 (19.3–28.0)
Tennessee	1,630,000	575,000	41.2 (38.0–44.5)	38.9 (33.7–44.3)
Texas	4,055,000	1,519,000	43.0 (40.0–46.0)	43.4 (38.5–48.5)
Utah	407,000	106,000	29.4 (27.2–31.6)	28.0 (24.8–31.4)
Vermont	136,000	40,000	32.1 (29.4–35.0)	31.0 (26.4–36.1)
Virginia	1,513,000	520,000	38.1 (35.4–40.9)	36.3 (31.9–40.9)
Washington	1,346,000	317,000	26.3 (24.6–28.0)	25.8 (22.6–29.4)
West Virginia	557,000	209,000	41.0 (38.7–43.4)	38.6 (35.0–42.3)
Wisconsin	1,104,000	281,000	29.0 (26.2–32.1)	24.6 (19.9–30.0)
Wyoming	116,000	37,000	35.2 (31.8–38.8)	34.4 (28.6–40.7)
*Median (Range)^¶^*			*37.1 (24.7–50.1)*	*35.0 (23.1–47.9)*
Guam	17,000	7,000	45.7 (37.1–54.5)	39.6 (29.8–50.2)
Puerto Rico	635,000	371,000	60.5 (57.5–63.5)	58.1 (52.1–63.8)

**Abbreviation**: CI = confidence interval.

* Age standardized to the 2000 U.S. projected population, using three age groups: 18–44, 45–64, and ≥65 years.

^†^ Physical inactivity was defined as a no response to the question “During the past month, other than your regular job, did you participate in any physical activities or exercises such as running, calisthenics, golf, gardening, or walking for exercise?”

^§^ Doctor-diagnosed arthritis was defined as a yes response to the question “Has a doctor, nurse, or other health professional ever told you that you have some form of arthritis, rheumatoid arthritis, gout, lupus, or fibromyalgia?”

^¶^ Median and range were calculated from estimates for the 50 states and the District of Columbia.

**TABLE 8:** Unadjusted and age-standardized* percentages of walking^†^ among adults aged ≥18 years with arthritis,^§^ by area — Behavioral Risk Factor Surveillance System, United States, 2015

Area	Weighted population with arthritis (rounded to 1,000s)	Weighted population with arthritis and walking (rounded to 1,000s)	Unadjusted % (95% CI)	Age-standardized % (95% CI)
Alabama	1,248,000	501,000	43.6 (41.1–46.0)	44.8 (40.7–48.9)
Alaska	117,000	59,000	53.9 (48.9–58.8)	52.3 (44.8–59.7)
Arizona	1,222,000	570,000	51.5 (48.7–54.3)	50.8 (45.3–56.2)
Arkansas	672,000	249,000	40.6 (37.0–44.3)	41.8 (35.4–48.4)
California	5,719,000	2,996,000	59.4 (56.7–62.0)	55.6 (51.3–59.9)
Colorado	949,000	464,000	55.4 (52.9–57.7)	55.4 (50.9–59.8)
Connecticut	690,000	308,000	49.4 (47.2–51.7)	51.5 (47.1–55.9)
Delaware	207,000	83,000	42.9 (39.4–46.4)	43.1 (36.5–49.9)
District of Columbia	101,000	50,000	53.6 (48.2–59.0)	51.9 (40.2–63.5)
Florida	4,154,000	1,751,000	47.2 (44.7–49.7)	46.9 (41.4–52.4)
Georgia	1,890,000	829,000	47.7 (44.4–51.1)	45.2 (38.8–51.7)
Hawaii	211,000	101,000	52.1 (48.8–55.4)	47.2 (41.4–53.1)
Idaho	309,000	157,000	54.1 (50.9–57.3)	53.0 (47.3–58.5)
Illinois	2,308,000	949,000	44.6 (41.5–47.7)	45.7 (39.9–51.6)
Indiana	1,390,000	615,000	48.3 (45.2–51.4)	51.8 (45.8–57.7)
Iowa	619,000	268,000	47.2 (44.4–50.1)	43.5 (38.1–49.0)
Kansas	536,000	230,000	47.4 (46.0–48.9)	48.9 (46.2–51.5)
Kentucky	1,087,000	426,000	42.9 (40.0–45.8)	46.2 (41.2–51.2)
Louisiana	989,000	377,000	42.3 (39.0–45.6)	46.3 (40.9–51.9)
Maine	332,000	158,000	50.7 (48.2–53.1)	51.0 (46.2–55.7)
Maryland	1,096,000	457,000	46.5 (43.5–49.6)	46.2 (40.2–52.3)
Massachusetts	1,300,000	539,000	46.9 (44.1–49.7)	43.4 (38.9–48.0)
Michigan	2,305,000	1,018,000	46.8 (44.6–49.0)	45.7 (42.1–49.3)
Minnesota	907,000	441,000	51.6 (49.7–53.5)	52.6 (48.9–56.2)
Mississippi	647,000	248,000	40.2 (37.4–43.1)	40.3 (35.3–45.5)
Missouri	1,372,000	592,000	46.1 (43.4–49.0)	48.0 (42.7–53.3)
Montana	216,000	120,000	57.9 (54.8–60.9)	59.5 (53.8–64.9)
Nebraska	334,000	161,000	51.0 (48.9–53.1)	52.9 (48.7–57.1)
Nevada	477,000	226,000	51.2 (45.5–56.9)	49.3 (39.9–58.9)
New Hampshire	282,000	129,000	49.9 (47.1–52.6)	49.1 (43.2–55.0)
New Jersey	1,590,000	670,000	45.3 (42.6–48.1)	43.6 (38.8–48.6)
New Mexico	386,000	185,000	52.5 (49.4–55.6)	52.4 (46.3–58.4)
New York	3,629,000	1,540,000	48.3 (46.1–50.6)	47.3 (43.0–51.6)
North Carolina	2,089,000	892,000	45.2 (42.5–47.9)	45.6 (41.1–50.2)
North Dakota	134,000	67,000	52.7 (49.3–56.0)	52.0 (45.5–58.4)
Ohio	2,547,000	1,068,000	45.3 (42.9–47.8)	48.2 (43.4–53.0)
Oklahoma	813,000	315,000	41.1 (38.4–43.8)	43.1 (38.3–48.0)
Oregon	838,000	413,000	54.2 (51.1–57.1)	53.7 (48.5–58.9)
Pennsylvania	2,937,000	1,234,000	46.3 (43.4–49.3)	44.4 (39.3–49.6)
Rhode Island	226,000	89,000	43.9 (41.0–46.9)	41.5 (35.6–47.5)
South Carolina	1,105,000	479,000	46.2 (44.1–48.3)	45.3 (41.3–49.2)
South Dakota	158,000	80,000	53.6 (50.0–57.2)	54.5 (47.9–61.0)
Tennessee	1,630,000	680,000	46.3 (43.2–49.5)	46.4 (41.1–51.8)
Texas	4,055,000	1,651,000	45.2 (42.3–48.2)	40.3 (36.1–44.7)
Utah	407,000	201,000	53.6 (51.3–56.0)	53.1 (49.6–56.7)
Vermont	136,000	69,000	53.3 (50.4–56.1)	53.5 (48.3–58.6)
Virginia	1,513,000	663,000	46.6 (44.0–49.3)	46.1 (41.2–51.0)
Washington	1,346,000	710,000	56.4 (54.5–58.3)	54.6 (50.7–58.5)
West Virginia	557,000	208,000	39.0 (36.8–41.3)	38.5 (35.0–42.1)
Wisconsin	1,104,000	544,000	54.4 (51.0–57.7)	57.0 (50.2–63.5)
Wyoming	116,000	54,000	50.2 (46.7–53.8)	50.0 (43.8–56.3)
*Median (Range)^¶^*			*47.7 (39.0–59.4)*	*48.0 (38.5–59.5)*
Guam	17,000	6,000	36.3 (28.6–44.7)	34.0 (23.5–46.3)
Puerto Rico	635,000	173,000	27.8 (25.2–30.6)	28.5 (23.4–34.1)

**Abbreviation**: CI = confidence interval.

* Age standardized to the 2000 U.S. projected population, using three age groups: 18–44, 45–64, and ≥65 years.

^†^ Respondents with arthritis who reported walking or hiking for one of two questions: 1) “What type of physical activity or exercise did you spend the most time doing during the past month?” and 2) “What other type of physical activity gave you the next most exercise during the past month?” The denominator included adults with arthritis who were either physically active or inactive.

^§^ Doctor-diagnosed arthritis was defined as a yes response to the question “Has a doctor, nurse, or other health professional ever told you that you have some form of arthritis, rheumatoid arthritis, gout, lupus, or fibromyalgia?”

^¶^ Median and range were calculated from estimates for the 50 states and the District of Columbia.

For the 50 states and the District of Columbia, the percentage of obesity among adults with arthritis varied substantially. The median age-standardized percentage of obesity was 41.6% (range: 28.1% in California to 48.9% in Arkansas) (Table 9).

**TABLE 9:** Unadjusted and age-standardized* percentages of obesity^†^ among adults aged ≥18 years with arthritis,^§^ by area — Behavioral Risk Factor Surveillance System, United States, 2015

Area	Weighted population with arthritis (rounded to 1,000s)	Weighted population with arthritis and obesity (rounded to 1,000s)	Unadjusted % (95% CI)	Age-standardized % (95% CI)
Alabama	1,248,000	512,000	43.8 (41.4–46.2)	45.9 (42.0–49.9)
Alaska	117,000	46,000	41.6 (36.9–46.5)	45.0 (37.8–52.4)
Arizona	1,222,000	398,000	35.6 (33.0–38.4)	34.6 (29.9–39.6)
Arkansas	672,000	275,000	44.2 (40.5–47.9)	48.9 (42.5–55.4)
California	5,719,000	1,749,000	33.0 (30.6–35.6)	33.7 (29.8–37.9)
Colorado	949,000	250,000	28.9 (26.8–31.1)	28.1 (24.7–31.9)
Connecticut	690,000	228,000	35.7 (33.6–37.9)	37.3 (33.2–41.6)
Delaware	207,000	70,000	37.5 (34.1–41.0)	38.4 (32.1–45.1)
District of Columbia	101,000	41,000	43.9 (38.5–49.5)	42.4 (30.7–55.0)
Florida	4,154,000	1,381,000	36.0 (33.6–38.5)	38.4 (33.2–43.8)
Georgia	1,890,000	759,000	42.2 (38.9–45.5)	38.0 (32.6–43.8)
Hawaii	211,000	67,000	32.5 (29.5–35.8)	42.2 (36.6–48.0)
Idaho	309,000	105,000	36.4 (33.3–39.6)	37.3 (32.1–42.8)
Illinois	2,308,000	946,000	42.1 (39.0–45.2)	41.8 (36.2–47.6)
Indiana	1,390,000	537,000	41.7 (38.7–44.7)	44.1 (38.3–50.1)
Iowa	619,000	241,000	42.2 (39.4–45.0)	44.7 (39.3–50.3)
Kansas	536,000	194,000	41.1 (39.7–42.6)	43.2 (40.6–45.9)
Kentucky	1,087,000	457,000	44.9 (42.0–47.9)	48.5 (43.5–53.5)
Louisiana	989,000	419,000	45.3 (42.0–48.6)	48.5 (43.2–53.9)
Maine	332,000	122,000	38.7 (36.3–41.1)	42.3 (37.7–47.0)
Maryland	1,096,000	448,000	44.4 (41.2–47.6)	47.9 (41.8–54.1)
Massachusetts	1,300,000	422,000	36.1 (33.4–38.9)	35.8 (31.5–40.3)
Michigan	2,305,000	875,000	40.4 (38.2–42.6)	42.1 (38.6–45.8)
Minnesota	907,000	321,000	38.3 (36.5–40.2)	40.3 (36.6–44.0)
Mississippi	647,000	260,000	42.0 (39.1–44.9)	44.3 (39.2–49.5)
Missouri	1,372,000	518,000	40.7 (38.0–43.5)	42.9 (37.8–48.2)
Montana	216,000	62,000	31.1 (28.3–34.1)	29.6 (24.8–34.8)
Nebraska	334,000	134,000	42.6 (40.6–44.7)	45.1 (40.9–49.2)
Nevada	477,000	155,000	34.4 (29.2–39.9)	35.2 (26.7–44.7)
New Hampshire	282,000	94,000	36.9 (34.3–39.6)	40.7 (35.0–46.6)
New Jersey	1,590,000	539,000	37.3 (34.6–40.1)	38.7 (34.1–43.6)
New Mexico	386,000	127,000	35.2 (32.2–38.3)	43.1 (37.3–49.1)
New York	3,629,000	1,221,000	36.4 (34.2–38.6)	40.1 (36.0–44.4)
North Carolina	2,089,000	780,000	40.0 (37.3–42.7)	41.4 (37.0–46.0)
North Dakota	134,000	51,000	41.3 (38.1–44.7)	39.7 (34.1–45.6)
Ohio	2,547,000	947,000	40.2 (37.8–42.7)	40.4 (35.7–45.2)
Oklahoma	813,000	329,000	42.9 (40.2–45.7)	44.9 (40.1–49.8)
Oregon	838,000	302,000	39.3 (36.4–42.4)	41.6 (36.6–46.8)
Pennsylvania	2,937,000	1,114,000	40.8 (37.9–43.7)	43.3 (38.2–48.5)
Rhode Island	226,000	74,000	35.1 (32.2–38.1)	34.7 (29.1–40.7)
South Carolina	1,105,000	437,000	42.0 (39.9–44.1)	46.4 (42.6–50.3)
South Dakota	158,000	60,000	40.4 (36.9–44.0)	41.8 (35.3–48.5)
Tennessee	1,630,000	640,000	42.3 (39.2–45.5)	46.5 (41.3–51.8)
Texas	4,055,000	1,518,000	41.0 (38.1–43.9)	37.6 (33.4–42.0)
Utah	407,000	137,000	36.2 (33.9–38.5)	36.2 (32.9–39.6)
Vermont	136,000	43,000	34.2 (31.5–36.9)	35.7 (30.8–40.9)
Virginia	1,513,000	584,000	41.1 (38.4–43.9)	44.0 (39.2–48.9)
Washington	1,346,000	454,000	36.9 (35.0–38.8)	36.5 (33.0–40.3)
West Virginia	557,000	223,000	43.7 (41.4–46.1)	45.6 (41.9–49.3)
Wisconsin	1,104,000	417,000	40.2 (37.0–43.5)	41.0 (34.9–47.4)
Wyoming	116,000	41,000	37.6 (34.2–41.1)	36.1 (30.6–42.0)
*Median (Range)^¶^*			*40.2 (28.9–45.3)*	*41.6 (28.1–48.9)*
Guam	17,000	7,000	39.3 (31.5–47.6)	42.6 (31.9–54.1)
Puerto Rico	635,000	215,000	35.6 (32.6–38.6)	41.1 (35.3–47.2)

**Abbreviation**: CI = confidence interval.

* Age standardized to the 2000 U.S. projected population, using three age groups: 18–44, 45–64, and ≥65 years.

^†^ Body mass index ≥30; calculated from self-reported height and weight.

^§^ Doctor-diagnosed arthritis was defined as a yes response to the question “Has a doctor, nurse, or other health professional ever told you that you have some form of arthritis, rheumatoid arthritis, gout, lupus, or fibromyalgia?”

^¶^ Median and range were calculated from estimates for the 50 states and the District of Columbia.

#### Activity Limitations

In 2015, for the 50 states and the District of Columbia, the median age-standardized percentage of arthritis-attributable activity limitations among adults with arthritis was 49.7% (range: 40.4% in Iowa to 59.4% in Missouri) (Table 10). The median age-standardized percentage for arthritis-attributable social participation restriction was 19.7% (range: 12.6% in Alaska to 30.4% in Arkansas) (Table 11).

**TABLE 10:** Unadjusted and age-standardized* percentages of arthritis-attributable activity limitations^†^ among adults aged ≥18 years with arthritis,^§^ by area — Behavioral Risk Factor Surveillance System, United States, 2015

Area	Weighted population with arthritis (rounded to 1,000s)	Weighted population with arthritis and arthritis-attributable activity limitations (rounded to 1,000s)	Unadjusted % (95% CI)	Age-standardized % (95% CI)
Alabama	1,248,000	640,000	56.7 (54.3–59.1)	59.2 (55.2–63.0)
Alaska	117,000	55,000	51.2 (46.2–56.1)	51.1 (43.6–58.6)
Arizona	1,222,000	558,000	52.1 (49.3–54.9)	52.5 (46.9–58.0)
Arkansas	672,000	345,000	57.0 (53.2–60.7)	56.1 (49.4–62.5)
California	5,719,000	2,446,000	49.6 (46.9–52.3)	48.6 (44.2–52.9)
Colorado	949,000	398,000	48.4 (46.0–50.8)	47.6 (43.1–52.1)
Connecticut	690,000	290,000	47.6 (45.3–49.8)	46.7 (42.3–51.2)
Delaware	207,000	90,000	47.1 (43.5–50.8)	46.9 (39.9–54.0)
District of Columbia	101,000	48,000	53.9 (48.4–59.3)	57.0 (45.0–68.2)
Florida	4,154,000	1,941,000	53.1 (50.6–55.6)	54.5 (48.9–59.9)
Georgia	1,890,000	882,000	51.9 (48.4–55.3)	50.9 (44.1–57.6)
Hawaii	211,000	81,000	42.2 (39.0–45.5)	42.0 (36.2–48.0)
Idaho	309,000	137,000	48.0 (44.8–51.2)	50.4 (44.8–56.0)
Illinois	2,308,000	982,000	46.5 (43.3–49.6)	46.3 (40.5–52.2)
Indiana	1,390,000	599,000	47.7 (44.6–50.8)	43.7 (38.1–49.5)
Iowa	619,000	228,000	40.8 (38.1–43.6)	40.4 (34.9–46.0)
Kansas	536,000	228,000	48.2 (46.7–49.7)	46.3 (43.7–49.0)
Kentucky	1,087,000	562,000	57.2 (54.3–60.0)	57.7 (52.7–62.6)
Louisiana	989,000	472,000	53.9 (50.6–57.2)	50.6 (45.0–56.1)
Maine	332,000	149,000	48.5 (46.0–50.9)	51.2 (46.4–56.0)
Maryland	1,096,000	405,000	42.2 (39.2–45.2)	42.2 (36.3–48.4)
Massachusetts	1,300,000	555,000	49.0 (46.2–51.9)	47.0 (42.2–51.9)
Michigan	2,305,000	1,038,000	48.8 (46.6–51.1)	50.3 (46.5–54.0)
Minnesota	907,000	393,000	46.7 (44.8–48.5)	47.0 (43.3–50.6)
Mississippi	647,000	346,000	56.7 (53.7–59.6)	52.4 (47.2–57.4)
Missouri	1,372,000	694,000	54.6 (51.8–57.4)	59.4 (54.5–64.1)
Montana	216,000	103,000	50.2 (47.0–53.4)	52.0 (46.3–57.8)
Nebraska	334,000	137,000	44.0 (41.9–46.1)	44.3 (40.1–48.6)
Nevada	477,000	212,000	48.7 (43.0–54.5)	53.3 (44.2–62.3)
New Hampshire	282,000	115,000	45.6 (42.8–48.3)	49.9 (43.8–55.9)
New Jersey	1,590,000	675,000	46.5 (43.8–49.3)	47.0 (42.0–52.2)
New Mexico	386,000	176,000	50.5 (47.4–53.6)	49.2 (43.1–55.4)
New York	3,629,000	1,503,000	48.3 (46.0–50.6)	45.5 (41.2–49.9)
North Carolina	2,089,000	1,080,000	55.6 (52.9–58.3)	54.5 (49.9–59.0)
North Dakota	134,000	57,000	46.3 (42.9–49.6)	47.2 (40.5–53.9)
Ohio	2,547,000	1,136,000	49.1 (46.6–51.6)	46.6 (42.0–51.3)
Oklahoma	813,000	419,000	55.9 (53.2–58.7)	56.9 (51.9–61.7)
Oregon	838,000	425,000	57.1 (54.1–60.1)	54.0 (48.8–59.2)
Pennsylvania	2,937,000	1,118,000	42.7 (39.8–45.7)	42.1 (37.0–47.4)
Rhode Island	226,000	89,000	45.1 (42.0–48.2)	44.1 (37.6–50.7)
South Carolina	1,105,000	555,000	54.8 (52.7–56.9)	54.4 (50.3–58.4)
South Dakota	158,000	73,000	48.7 (45.1–52.4)	49.5 (42.7–56.3)
Tennessee	1,630,000	772,000	53.8 (50.6–57.0)	55.2 (49.8–60.5)
Texas	4,055,000	1,773,000	50.0 (47.0–53.0)	48.2 (43.3–53.1)
Utah	407,000	168,000	45.5 (43.1–47.8)	44.2 (40.8–47.8)
Vermont	136,000	61,000	49.0 (46.2–51.9)	48.6 (43.4–53.8)
Virginia	1,513,000	631,000	44.8 (42.2–47.5)	46.7 (41.9–51.6)
Washington	1,346,000	657,000	53.3 (51.4–55.2)	50.6 (46.6–54.5)
West Virginia	557,000	303,000	57.3 (55.0–59.6)	57.5 (53.8–61.2)
Wisconsin	1,104,000	479,000	49.2 (45.9–52.7)	50.9 (44.1–57.7)
Wyoming	116,000	49,000	46.6 (43.0–50.3)	49.7 (43.4–55.9)
*Median (Range)^¶^*			*49.0 (40.8–57.3)*	*49.7 (40.4–59.4)*
Guam	17,000	7,000	43.5 (35.4–52.0)	44.6 (33.4–56.3)
Puerto Rico	635,000	353,000	57.0 (54.0–60.0)	60.8 (54.9–66.4)

**Abbreviation:** CI = confidence interval.

* Age standardized to the 2000 U.S. projected population, using three age groups: 18–44, 45–64, and ≥65 years.

^†^ Respondents with arthritis who answered yes to the question “Arthritis can cause symptoms like pain, aching, or stiffness in or around the joint. Are you now limited in any way in any of your usual activities because of arthritis or joint symptoms?”

^§^ Doctor-diagnosed arthritis was defined as a yes response to the question “Has a doctor, nurse, or other health professional ever told you that you have some form of arthritis, rheumatoid arthritis, gout, lupus, or fibromyalgia?”

*^¶^* Median and range were calculated from estimates for the 50 states and the District of Columbia.

**TABLE 11:** Unadjusted and age-standardized* percentages of arthritis-attributable social participation restriction^†^ among adults aged ≥18 years with arthritis,^§^ by area — Behavioral Risk Factor Surveillance System, United States, 2015

Area	Weighted population with arthritis (rounded to 1,000s)	Weighted population with arthritis and arthritis-attributable social participation restriction (rounded to 1,000s)	Unadjusted % (95% CI)	Age-standardized % (95% CI)
Alabama	1,248,000	321,000	28.5 (26.3–30.9)	29.3 (25.8–33.0)
Alaska	117,000	16,000	14.4 (11.3–18.3)	12.6 (9.3–16.9)
Arizona	1,222,000	212,000	19.7 (17.6–22.0)	18.1 (14.8–22.1)
Arkansas	672,000	165,000	27.3 (24.1–30.9)	30.4 (24.5–37.0)
California	5,719,000	859,000	17.4 (15.6–19.4)	17.6 (14.6–21.0)
Colorado	949,000	128,000	15.5 (13.7–17.4)	16.4 (13.4–20.1)
Connecticut	690,000	97,000	15.9 (14.2–17.8)	14.5 (12.1–17.4)
Delaware	207,000	35,000	18.5 (15.7–21.6)	18.8 (13.4–25.8)
District of Columbia	101,000	22,000	25.2 (20.4–30.7)	26.8 (17.4–38.8)
Florida	4,154,000	843,000	23.3 (21.1–25.6)	25.7 (21.0–31.1)
Georgia	1,890,000	378,000	22.3 (19.5–25.3)	19.8 (15.3–25.3)
Hawaii	211,000	26,000	13.4 (11.1–16.0)	15.9 (11.4–21.7)
Idaho	309,000	53,000	18.7 (16.2–21.6)	22.4 (17.7–28.0)
Illinois	2,308,000	365,000	17.4 (15.0–20.1)	15.0 (11.7–19.0)
Indiana	1,390,000	237,000	18.8 (16.6–21.4)	20.0 (15.7–25.2)
Iowa	619,000	83,000	14.9 (13.0–17.0)	15.3 (11.6–19.9)
Kansas	536,000	84,000	17.6 (16.5–18.8)	17.6 (15.7–19.6)
Kentucky	1,087,000	265,000	27.1 (24.6–29.9)	28.3 (23.6–33.6)
Louisiana	989,000	234,000	26.8 (23.9–29.8)	25.5 (20.8–30.7)
Maine	332,000	52,000	16.9 (15.1–18.9)	21.4 (17.5–25.9)
Maryland	1,096,000	160,000	16.6 (14.4–19.0)	19.7 (14.9–25.7)
Massachusetts	1,300,000	206,000	18.2 (16.1–20.6)	20.0 (16.2–24.5)
Michigan	2,305,000	420,000	19.6 (17.8–21.5)	19.6 (16.8–22.7)
Minnesota	907,000	136,000	16.1 (14.7–17.7)	18.5 (15.5–22.0)
Mississippi	647,000	172,000	28.5 (25.8–31.3)	26.0 (22.1–30.4)
Missouri	1,372,000	255,000	20.2 (18.0–22.5)	23.4 (19.2–28.3)
Montana	216,000	33,000	16.2 (13.8–18.8)	18.6 (14.2–24.0)
Nebraska	334,000	46,000	14.6 (13.2–16.2)	12.9 (10.7–15.6)
Nevada	477,000	98,000	22.7 (17.9–28.3)	26.0 (17.6–36.6)
New Hampshire	282,000	40,000	15.7 (13.8–17.9)	20.2 (15.6–25.8)
New Jersey	1,590,000	241,000	16.8 (14.9–18.9)	18.5 (15.2–22.3)
New Mexico	386,000	71,000	20.6 (18.1–23.2)	21.0 (16.5–26.4)
New York	3,629,000	587,000	19.0 (17.2–20.9)	19.8 (16.4–23.7)
North Carolina	2,089,000	488,000	25.0 (22.7–27.6)	23.7 (20.1–27.6)
North Dakota	134,000	18,000	14.7 (12.5–17.3)	14.8 (10.8–19.9)
Ohio	2,547,000	486,000	21.1 (19.1–23.2)	19.7 (16.6–23.2)
Oklahoma	813,000	207,000	27.5 (24.9–30.3)	29.9 (25.4–34.7)
Oregon	838,000	142,000	19.0 (16.6–21.6)	19.4 (15.7–23.7)
Pennsylvania	2,937,000	419,000	16.0 (13.9–18.4)	16.8 (13.0–21.3)
Rhode Island	226,000	39,000	20.0 (17.4–22.9)	25.8 (20.0–32.6)
South Carolina	1,105,000	245,000	24.2 (22.4–26.1)	23.8 (20.5–27.5)
South Dakota	158,000	20,000	13.7 (11.3–16.5)	14.4 (9.5–21.1)
Tennessee	1,630,000	372,000	26.0 (23.2–28.9)	24.7 (20.3–29.6)
Texas	4,055,000	739,000	20.8 (18.5–23.3)	20.7 (16.9–25.2)
Utah	407,000	58,000	15.6 (13.9–17.4)	16.0 (13.6–18.8)
Vermont	136,000	21,000	16.3 (14.1–18.7)	18.9 (14.9–23.6)
Virginia	1,513,000	251,000	17.8 (16.0–19.8)	18.1 (14.9–21.9)
Washington	1,346,000	221,000	17.8 (16.3–19.4)	18.0 (15.3–21.0)
West Virginia	557,000	140,000	26.7 (24.6–28.9)	26.6 (23.5–30.0)
Wisconsin	1,104,000	153,000	15.9 (13.6–18.4)	16.6 (12.8–21.1)
Wyoming	116,000	19,000	17.2 (14.3–20.6)	19.3 (14.1–25.8)
*Median (Range)^¶^*			*18.5 (13.4–28.5)*	*19.7 (12.6–30.4)*
Guam	17,000	2,000	12.7 (8.5–18.4)	13.1 (7.7–21.5)
Puerto Rico	635,000	144,000	23.2 (20.8–25.9)	25.9 (21.2–31.2)

**Abbreviation:** CI = confidence interval.

* Age standardized to the 2000 U.S. projected population, using three age groups: 18–44, 45–64, and ≥65 years.

^†^ Respondents with arthritis who answered a lot to the question “During the past 30 days, to what extent has your arthritis or joint symptoms interfered with your normal social activities, such as going shopping, to the movies, or to religious or social gatherings?”

^§^ Doctor-diagnosed arthritis was defined as a yes response to the question “Has a doctor, nurse, or other health professional ever told you that you have some form of arthritis, rheumatoid arthritis, gout, lupus, or fibromyalgia?”

^¶^ Median and range were calculated from estimates for the 50 states and the District of Columbia.

#### Pain

**FIGURE 4:**
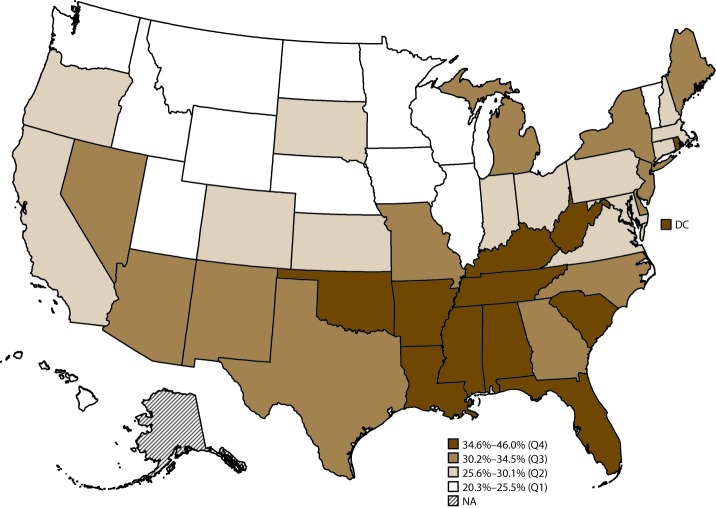
Age-standardized prevalence of arthritis-attributable severe joint pain* among adults aged ≥18 years with arthritis, by state^†^ — Behavioral Risk Factor Surveillance System, United States, 2015 **Abbreviations:** DC = District of Columbia; NA = not applicable; Q = quartile. * Respondents with arthritis who answered 7, 8, 9, or 10 to the question “Please think about the past 30 days, keeping in mind all of your joint pain or aching and whether or not you have taken medication. During the past 30 days, how bad was your joint pain on average? Please answer on a scale of 0 to 10 where 0 is no pain or aching and 10 is pain or aching as bad as it can be.” Severe joint pain was defined as a pain level of 7–10. ^†^ Estimate for one state (Alaska) with a relative standard error >30% or unweighted denominator <50 was suppressed as unreliable.

In 2015, the median age-standardized percentage of arthritis-attributable severe joint pain among adults with arthritis was 29.7% (range: 20.3% in Utah to 46.0% in Mississippi) (Table 12). States with the highest age-standardized percentage of arthritis-attributable severe joint pain among adults with arthritis tended be primarily in Appalachia and in the South (Figure 4).

**TABLE 12:** Unadjusted and age-standardized* percentages of arthritis-attributable severe joint pain^†^ among adults aged ≥18 years with arthritis,^§^ by area — Behavioral Risk Factor Surveillance System, United States, 2015

Area	Weighted population with arthritis (rounded to 1,000s)	Weighted population with arthritis and arthritis-attributable severe joint pain (rounded to 1,000s)	Unadjusted % (95% CI)	Age-standardized % (95% CI)
Alabama	1,248,000	433,000	39.2 (36.8–41.6)	39.7 (35.7–43.8)
Alaska	117,000	—^¶^	—^¶^	—^¶^
Arizona	1,222,000	328,000	30.6 (28.0–33.3)	32.9 (28.0–38.2)
Arkansas	672,000	218,000	36.3 (32.6–40.1)	41.6 (35.3–48.3)
California	5,719,000	1,510,000	30.2 (27.9–32.7)	29.3 (25.8–33.0)
Colorado	949,000	194,000	24.0 (21.9–26.2)	26.2 (22.3–30.4)
Connecticut	690,000	153,000	25.3 (23.3–27.4)	25.9 (22.3–29.8)
Delaware	207,000	57,000	30.8 (27.3–34.4)	31.6 (25.2–38.7)
District of Columbia	101,000	36,000	40.5 (35.0–46.3)	36.5 (26.5–47.9)
Florida	4,154,000	1,195,000	34.0 (31.5–36.6)	38.0 (32.7–43.7)
Georgia	1,890,000	573,000	34.1 (30.8–37.4)	31.2 (25.7–37.4)
Hawaii	211,000	41,000	21.7 (19.0–24.7)	23.6 (18.9–29.2)
Idaho	309,000	59,000	21.8 (19.1–24.9)	21.9 (17.4–27.3)
Illinois	2,308,000	576,000	27.4 (24.6–30.5)	24.4 (20.2–29.3)
Indiana	1,390,000	302,000	25.5 (22.8–28.4)	26.0 (20.6–32.2)
Iowa	619,000	122,000	22.3 (20.0–24.8)	24.3 (19.7–29.6)
Kansas	536,000	121,000	25.7 (24.4–27.1)	26.2 (23.8–28.6)
Kentucky	1,087,000	349,000	36.3 (33.5–39.1)	36.7 (32.0–41.7)
Louisiana	989,000	356,000	41.1 (37.8–44.4)	41.2 (35.8–46.9)
Maine	332,000	69,000	23.9 (21.6–26.3)	30.3 (25.6–35.4)
Maryland	1,096,000	249,000	26.1 (23.3–29.1)	28.5 (22.8–35.0)
Massachusetts	1,300,000	310,000	28.0 (25.4–30.8)	28.4 (24.1–33.2)
Michigan	2,305,000	605,000	28.7 (26.7–30.8)	30.5 (27.1–34.1)
Minnesota	907,000	181,000	21.8 (20.1–23.5)	23.3 (19.9–27.1)
Mississippi	647,000	270,000	45.5 (42.5–48.5)	46.0 (40.8–51.3)
Missouri	1,372,000	375,000	30.1 (27.6–32.8)	34.0 (29.2–39.2)
Montana	216,000	47,000	23.2 (20.5–26.1)	24.3 (19.7–29.6)
Nebraska	334,000	67,000	21.6 (19.9–23.5)	22.8 (19.4–26.6)
Nevada	477,000	130,000	30.6 (25.6–36.1)	30.2 (22.3–39.3)
New Hampshire	282,000	58,000	23.0 (20.7–25.6)	26.6 (21.6–32.3)
New Jersey	1,590,000	424,000	30.0 (27.5–32.6)	33.6 (28.9–38.7)
New Mexico	386,000	110,000	32.2 (29.3–35.2)	32.7 (27.4–38.5)
New York	3,629,000	821,000	30.9 (28.6–33.3)	32.3 (27.8–37.2)
North Carolina	2,089,000	687,000	35.8 (33.2–38.6)	34.5 (30.3–38.9)
North Dakota	134,000	21,000	19.2 (16.3–22.4)	23.4 (17.8–30.2)
Ohio	2,547,000	695,000	30.4 (28.1–32.8)	30.1 (25.8–34.6)
Oklahoma	813,000	259,000	35.0 (32.3–37.9)	36.9 (32.2–41.9)
Oregon	838,000	178,000	24.3 (21.6–27.2)	25.7 (21.2–30.8)
Pennsylvania	2,937,000	723,000	28.1 (25.5–30.9)	28.7 (23.9–34.0)
Rhode Island	226,000	59,000	29.9 (27.1–33.0)	34.7 (28.6–41.4)
South Carolina	1,105,000	361,000	36.1 (34.0–38.2)	36.7 (32.9–40.7)
South Dakota	158,000	36,000	24.1 (20.9–27.6)	27.3 (21.4–34.2)
Tennessee	1,630,000	481,000	34.2 (31.1–37.4)	35.3 (30.1–40.8)
Texas	4,055,000	1,138,000	33.3 (30.5–36.3)	32.0 (27.5–36.9)
Utah	407,000	77,000	21.2 (19.4–23.2)	20.3 (17.7–23.2)
Vermont	136,000	29,000	23.2 (20.8–25.9)	25.5 (21.0–30.7)
Virginia	1,513,000	381,000	27.5 (25.2–29.9)	26.7 (23.0–30.7)
Washington	1,346,000	282,000	23.0 (21.3–24.6)	22.7 (19.6–26.1)
West Virginia	557,000	204,000	39.8 (37.5–42.1)	41.6 (37.9–45.3)
Wisconsin	1,104,000	232,000	24.0 (21.3–27.0)	23.7 (19.1–28.9)
Wyoming	116,000	22,000	20.7 (17.7–24.2)	22.7 (17.3–29.3)
Median (Range)**			*28.4 (19.2–45.5)*	*29.7 (20.3–46.0)*
Guam	17,000	5,000	31.3 (24.0–39.6)	27.0 (19.8–35.8)
Puerto Rico	635,000	340,000	56.0 (52.9–59.0)	58.2 (52.1–64.1)

**Abbreviation:** CI = confidence interval.

* Age standardized to the 2000 U.S. projected population, using three age groups: 18–44, 45–64, and ≥65 years.

^†^ Respondents with arthritis who answered 7, 8, 9, or 10 to the question “Please think about the past 30 days, keeping in mind all of your joint pain or aching and whether or not you have taken medication. During the past 30 days, how bad was your joint pain on average? Please answer on a scale of 0 to 10 where 0 is no pain or aching and 10 is pain or aching as bad as it can be.” Severe joint pain was defined as a pain level of 7–10.

^§^ Doctor-diagnosed arthritis was defined as a yes response to the question “Has a doctor, nurse, or other health professional ever told you that you have some form of arthritis, rheumatoid arthritis, gout, lupus, or fibromyalgia?”

^¶^ Estimates with a relative standard error ≥30% or unweighted denominator <50 were suppressed as unreliable.

** Median and range were calculated from estimates for the 50 states and the District of Columbia.

### Arthritis Management

In 2015, among adults with arthritis in 13 states that included the BRFSS arthritis management module, the age-standardized median percentage of attendance at a self-management education course was 14.5% (range: 9.1% in New York to 19.0% in Montana) (Table 13). The median age-standardized percentage of health care provider counseling to lose weight if overweight or obese was 44.5% (range: 35.1% in Montana to 53.2% in New York) (Table 14). The age-standardized percentage of health care provider counseling for physical activity or exercise did not vary considerably among states (median: 58.5%; range: 52.3%–61.9%) (Table 15).

**TABLE 13:** Unadjusted* and age-standardized^†^ percentages of adults aged ≥18 years with arthritis^§^ reporting they attended a self-management education course for their arthritis,^¶^ by selected state — Behavioral Risk Factor Surveillance System, 13 states,** 2015

State	No. of respondents with arthritis	Weighted population with arthritis and attendance at self-management education course (rounded to 1,000s)	Unadjusted % (95% CI)	Age-standardized % (95% CI)
California	2,803	818,000	15.4 (11.5–20.3)	13.6 (9.3–19.6)
Kansas	7,320	53,000	11.2 (9.9–12.7)	11.9 (9.5–14.8)
Kentucky	3,565	110,000	11.5 (9.9–13.3)	12.2 (9.5–15.5)
Michigan	3,224	259,000	12.2 (10.4–14.1)	12.5 (9.3–16.5)
Minnesota	4,666	116,000	14.2 (12.9–15.5)	15.3 (12.7–18.4)
Missouri	2,808	157,000	13.5 (11.7–15.5)	14.5 (11.2–18.7)
Montana	2,123	29,000	14.5 (12.2–17.0)	19.0 (14.4–24.6)
New York	3,921	323,000	10.6 (8.7–12.8)	9.1 (6.9–11.9)
Oregon	1,828	130,000	17.2 (13.8–21.3)	18.1 (12.4–25.5)
Pennsylvania	2,059	294,000	11.9 (10.0–14.1)	15.1 (11.3–20.0)
Rhode Island	2,244	21,000	10.8 (9.1–12.8)	10.6 (7.7–14.4)
South Carolina	4,405	126,000	12.9 (11.4–14.5)	16.5 (13.2–20.4)
Utah	2,929	60,000	16.6 (13.9–19.8)	18.0 (13.5–23.5)
*Median (Range)^††^*			*12.9 (10.6–17.2)*	*14.5 (9.1–19.0)*

**Abbreviation:** CI = confidence interval.

* The numerator was the estimated number of adults with arthritis who reported ever having taken an educational course or class to learn to manage their arthritis. The denominator was the estimated number of adults with arthritis.

^†^ Age standardized to the 2000 U.S. projected population, using three age groups: 18–44, 45–64, and ≥65 years.

^§^ Doctor-diagnosed arthritis was defined as a yes response to the question “Has a doctor, nurse, or other health professional ever told you that you have some form of arthritis, rheumatoid arthritis, gout, lupus, or fibromyalgia?”

^¶^ Respondents who answered yes to the question “Have you ever taken an educational course or class to teach you how to manage problems related to your arthritis or joint symptoms?”

** States that administered the Behavioral Risk Factor Surveillance System (BRFSS) arthritis management module: California, Kansas, Kentucky, Michigan, Minnesota, Missouri, Montana, New York, Oregon, Pennsylvania, Rhode Island, South Carolina, and Utah.

^††^ Median and range were calculated from estimates for the 13 states that administered the BRFSS arthritis management module.

**TABLE 14:** Unadjusted* and age-standardized^†^ percentages of overweight or obesity^§^ among adults aged ≥18 years with arthritis^¶^ reporting health care provider counseling to lose weight to help with their arthritis or joint symptoms,** by selected state — Behavioral Risk Factor Surveillance System, 13 states,^††^ 2015

State	No. of respondents with arthritis	No. of respondents who are overweight or obese	Weighted population with arthritis who are overweight or obese (rounded to 1,000s)	Weighted population with arthritis who are overweight or obese reporting counseling to lose weight (rounded to 1,000s)	Unadjusted % (95% CI)	Age-standardized %* (95% CI)
California	2,803	504	5,719,000	1,762,000	48.9 (41.5–56.4)	40.1 (30.9–50.1)
Kansas	7,320	2,127	536,000	133,000	41.6 (39.0–44.1)	39.9 (35.7–44.3)
Kentucky	3,565	2,289	1,087,000	339,000	48.4 (44.9–52.0)	52.7 (46.6–58.7)
Michigan	3,224	1,375	2,305,000	734,000	48.6 (45.3–52.0)	49.4 (43.5–55.3)
Minnesota	4,666	2,885	907,000	246,000	43.0 (40.7–45.3)	44.3 (39.7–49.0)
Missouri	2,808	1,751	1,372,000	384,000	47.7 (44.4–51.0)	49.8 (43.0–56.6)
Montana	2,123	1,304	216,000	50,000	39.3 (35.6–43.0)	35.1 (28.7–42.1)
New York	39,21	1,112	3,629,000	1,118,000	52.9 (48.7–57.0)	53.2 (45.2–61.1)
Oregon	1,828	528	838,000	245,000	47.8 (42.0–53.7)	47.5 (36.7–58.5)
Pennsylvania	2,059	1,268	2,937,000	801,000	44.6 (41.1–48.2)	42.4 (35.7–49.4)
Rhode Island	2,244	1,271	226,000	64,000	49.9 (46.2–53.6)	41.5 (34.7–48.6)
South Carolina	4,405	2,812	1,105,000	327,000	46.6 (44.0–49.2)	47.6 (42.9–52.4)
Utah	2,929	919	407,000	114,000	45.6 (41.2–50.2)	44.5 (37.6–51.5)
*Median (Range)^§§^*					*47.7 (39.3–52.9)*	*44.5 (35.1–53.2)*

**Abbreviation:** CI = confidence interval.

* The numerator was the estimated number of adults with arthritis who reported being told by their doctor to exercise or get physical activity. The denominator was the estimated number of adults with arthritis.

^†^ Age standardized to the 2000 U.S. projected population, using three age groups: 18–44, 45–64, and ≥65 years.

^§^ Overweight: body mass index 25.0–29.9; obese: body mass index ≥30; calculated from self-reported height and weight.

^¶^ Doctor-diagnosed arthritis was defined as a yes response to the question “Has a doctor, nurse, or other health professional ever told you that you have some form of arthritis, rheumatoid arthritis, gout, lupus, or fibromyalgia?”

** Respondents who were overweight or obese who answered yes to the question “Has a doctor or other health professional ever suggested losing weight to help your arthritis or joint symptoms?”

^††^ States that administered the Behavioral Risk Factor Surveillance System (BRFSS) arthritis management module: California, Kansas, Kentucky, Michigan, Minnesota, Missouri, Montana, New York, Oregon, Pennsylvania, Rhode Island, South Carolina, and Utah.

^§§^ Median and range were calculated from estimates for the 13 states that administered the BRFSS arthritis management module.

**TABLE 15:** Unadjusted* and age-standardized^†^ percentages of adults aged ≥18 years with arthritis^§^ reporting health care provider counseling for physical activity or exercise,^¶^ by selected state — Behavioral Risk Factor Surveillance System, 13 states,** 2015

State	No. of respondents with arthritis	Weighted population with arthritis reporting counseling for physical activity or exercise (rounded to 1,000s)	Unadjusted % (95% CI)	Age standardized %* (95% CI)
California	2,803	3,022,000	57.0 (50.9–62.9)	57.8 (48.2–66.9)
Kansas	7,320	247,000	52.6 (50.4–54.7)	52.3 (48.4–56.1)
Kentucky	3,565	514,000	53.8 (50.8–56.8)	53.6 (48.3–58.8)
Michigan	3,224	1,242,000	59.0 (56.2–61.7)	61.5 (56.5–66.3)
Minnesota	4,666	462,000	57.1 (55.2–59.0)	58.1 (54.4–61.7)
Missouri	2,808	668,000	57.8 (54.9–60.6)	57.0 (51.1–62.8)
Montana	2,123	112,000	55.5 (52.3–58.6)	57.8 (52.0–63.4)
New York	3,921	1,925,000	63.4 (60.1–66.5)	61.9 (55.1–68.2)
Oregon	1,828	445,000	59.6 (54.6–64.4)	61.7 (52.9–69.9)
Pennsylvania	2,059	1,439,000	58.5 (55.4–61.4)	59.8 (54.0–65.4)
Rhode Island	2,244	115,000	60.9 (57.8–63.8)	59.6 (53.0–65.8)
South Carolina	4,405	586,000	60.4 (58.3–62.6)	61.2 (57.1–65.1)
Utah	2,929	207,000	58.2 (54.4–61.8)	58.5 (52.5–64.3)
*Median (Range)^††^*			*58.2 (52.6–63.4)*	*58.5 (52.3–61.9)*

**Abbreviation**: CI = confidence interval.

* The numerator was the estimated number of adults with arthritis who report being told by their health care provider to exercise or get physical activity. The denominator was the estimated number of adults with arthritis.

^†^ Age standardized to the 2000 U.S. projected population, using three age groups: 18–44, 45–64, and ≥65 years.

^§^ Doctor-diagnosed arthritis was defined as a yes response to the question “Has a doctor, nurse, or other health professional ever told you that you have some form of arthritis, rheumatoid arthritis, gout, lupus, or fibromyalgia?”

^¶^ Respondents who answered yes to the question “Has a doctor or other health professional ever suggested physical activity or exercise to help your arthritis or joint symptoms?”

** States that administered the Behavioral Risk Factor Surveillance System (BRFSS) arthritis management module: California, Kansas, Kentucky, Michigan, Minnesota, Missouri, Montana, New York, Oregon, Pennsylvania, Rhode Island, South Carolina, and Utah.

^††^ Median and range were calculated from estimates for the 13 states that administered the BRFSS arthritis management module.

### Possible State-Specific Clustering of Health-Related Characteristics

States in the highest quartile of prevalence for adults with arthritis also had the highest percentages of all seven negative health-related characteristics (arthritis-attributable activity limitations, severe joint pain, and social participation restriction; ≥14 physically unhealthy days during the past 30 days; ≥14 mentally unhealthy days during the past 30 days; obesity; and leisure-time physical inactivity) and the lowest percentages of leisure-time walking compared with states in the lower quartiles (p-trend <0.006 for all characteristics) when both linear and quadratic tests for trends were conducted (Table 16). States in the highest quartile were Maine, Pennsylvania, West Virginia, Kentucky, Tennessee, Arkansas, Missouri, Oklahoma, Louisiana, Mississippi, Alabama, South Carolina, and Michigan.

**TABLE 16:** Estimates of adults aged ≥18 years with arthritis and age-standardized* percentages of arthritis health-related characteristics among adults with arthritis,^†^ by quartile^§^ of state-level prevalence of arthritis — Behavioral Risk Factor Surveillance System, United States, 2015

Estimate/Characteristic	Q1 (17.2%–21.5%) % (95% CI)	Q2 (21.5%–22.7%) % (95% CI)	Q3 (23.0%–25.4%) % (95% CI)	Q4 (25.7%–33.6%) % (95% CI)	p-trend^¶^
No. of respondents with arthritis	36,278	43,596	29,347	36,929	—
Weighted population with arthritis	21,702,000	12,741,000	10,866,000	15,694,000	—
Arthritis-attributable activity limitations	48.7 (46.7–50.6)	47.8 (46.1–49.5)	49.1 (47.1–51.2)	52.9 (51.3–54.4)	<0.001
Arthritis-attributable severe joint pain	31.3 (29.5–33.2)	26.6 (25.2–28.1)	29.5 (27.7–31.5)	35.4 (33.9–36.8)	<0.001
Arthritis-attributable social participation restriction	20.1 (18.5–21.8)	17.4 (16.2–18.6)	20.5 (18.9–22.1)	23.7 (22.5–25.1)	<0.001
≥14 physically unhealthy days during past 30 days	27.3 (25.6–29.0)	26.2 (24.7–27.7)	27.0 (25.3–28.8)	30.2 (28.9–31.5)	<0.006
≥14 mentally unhealthy days during past 30 days	22.6 (21.0–24.3)	22.1 (20.7–23.5)	24.0 (22.2–25.9)	25.7 (24.4–27.1)	0.001
Obesity	37.4 (35.6–39.3)	39.5 (37.8–41.2)	40.3 (38.4–42.4)	45.0 (43.6–46.5)	<0.001
Leisure-time physical inactivity	33.8 (32.0–35.8)	31.4 (29.8–33.0)	35.0 (33.0–37.0)	38.4 (37.0–39.9)	<0.001
Leisure-time walking	48.2 (46.3–50.1)	49.5 (47.8–51.2)	48.0 (46.0–50.1)	45.1 (43.6–46.6)	0.001

**Abbreviations:** CI = confidence interval; Q = quartile.

* Age standardized to the 2000 U.S. projected population, using three age groups: 18–44, 45–64, and ≥65 years.

^†^ Doctor-diagnosed arthritis was defined as a yes response to the question “Has a doctor, nurse, or other health professional ever told you that you have some form of arthritis, rheumatoid arthritis, gout, lupus, or fibromyalgia?”

^§^ Quartiles (Q1–Q4) were calculated from age-standardized prevalences of arthritis for the 50 states and the District of Columbia. Q1 (lowest): Hawaii, California, Minnesota, Texas, the District of Columbia, Nevada, New Jersey, Utah, Alaska, Nebraska, Maryland, New York, Florida, and Illinois. Q2: Virginia, North Dakota, Connecticut, Colorado, South Dakota, Arizona, Massachusetts, Wisconsin, New Mexico, Washington, and Kansas. Q3: New Hampshire, Iowa, Idaho, Vermont, Georgia, Montana, Wyoming, Rhode Island, Oregon, Delaware, North Carolina, Ohio, and Indiana. Q4 (highest): Pennsylvania, Oklahoma, Louisiana, South Carolina, Maine, Mississippi, Missouri, Michigan, Arkansas, Kentucky, Tennessee, Alabama, and West Virginia.

^¶^ p-value for test of linear trend in age-standardized prevalence estimates across quartiles. Bonferroni-corrected alpha level of 0.006 (α = 0.05/8) to adjust for testing multiple characteristics. Quadratic terms were applied to the test of trend to improve fit and were statistically significant at the alpha level of 0.006 for three characteristics (arthritis-attributable severe joint pain, arthritis-attributable social participation restriction, and leisure-time physical inactivity).

## Discussion

This is the first report of state-level arthritis prevalence estimates. The large sample size allowed precise estimates for even limited areas and analysis of health-related characteristics and comorbidities. This report also provides model-based county-level arthritis prevalence estimates with high internal validity, which help improve understanding of arthritis disparities at a local level.

In 2015, arthritis affected approximately one in four adults in the United States overall but prevalence, including model-based estimates at the county level, varied substantially by geographic area. The percentage of negative health-related characteristics among adults with arthritis was high in every area, but also varied substantially by geographic area. Arthritis management measures by state indicated both wide variation (e.g., health care provider counseling to lose weight if overweight or obese) and moderate variation (e.g., individual report of ever attending a self-management course and health care provider counseling for exercise or physical activity). Geographic disparities exist across the United States, with arthritis having the greatest impact in southern states (e.g., West Virginia, Kentucky, Tennessee, Arkansas, Missouri, Oklahoma, Louisiana, Mississippi, Alabama, and South Carolina). More detailed estimates for each of the 50 states, the District of Columbia, Guam, and Puerto Rico, along with additional analyses not reported here are available on the CDC Arthritis Program website (https://www.cdc.gov/arthritis/data_statistics/state-data-list-current.htm).

States with greater prevalences of arthritis also had greater percentages of negative health-related characteristics (i.e., arthritis-attributable activity limitations, arthritis-attributable severe joint pain, and arthritis-attributable social participation restriction; ≥14 physically unhealthy days during the past 30 days; ≥14 mentally unhealthy days during the past 30 days; obesity; and physical inactivity) and lesser percentages of leisure-time walking (a recommended management strategy) among adults with arthritis. The reasons for this geographic clustering are unknown but suggest a greater arthritis impact among adults with arthritis who live in those states. Geographic variation in four recognized risk factors for arthritis (i.e., obesity, occupations with high physical workload, smoking, and socioeconomic status) (*13*–*17*) that are also associated with negative health consequences among adults with arthritis might account for some of the difference. Geographic variations also might exist in access to medical care, including medications, resources for physical activity, and self-management interventions. Furthermore, because those states also have greater prevalence of coronary heart disease and diabetes, two important comorbid conditions for arthritis, health care providers might focus more on treatment and management of those chronic conditions with less emphasis on treatment and management of arthritis (*18*).

Adults with arthritis have a complex combination of disease characteristics and negative health consequences that can limit their daily activities; reduce health-related quality of life; and contribute to sustained obesity, leisure-time physical inactivity, and lack of participation in leisure-time walking. Participation in self-management education courses among adults with arthritis remains low (*19*). Only half of patients with arthritis receive counseling on the self-management behaviors of physical activity and weight loss. More counseling might help reduce the proportion with arthritis reporting obesity or leisure-time physical inactivity (approximately two in five adults) (*20*,*21*). Greater use of evidence-based interventions for physical activity and self-management education could reduce pain and improve function and quality of life for all adults with arthritis (*22*,*23*).

Nationally, approximately one in four adults with arthritis reported severe joint pain in the National Health Interview Survey (*4*); however, the geographic variations in this report suggest that the prevalence is higher in certain states (four in 10 might experience severe joint pain). Arthritis-attributable severe joint pain can lead to poor physical function. In a cohort of retirees in the United States with arthritis, approximately three in four reported functional limitations and approximately 65% had mobility limitations (*24*). Poor physical function is a major risk factor linked to falls (*25*), and adults with arthritis are more than twice as likely to report fall injuries compared with adults without arthritis (*26*). In addition to decrements in physical function, adults with arthritis consistently report negative effects on health-related quality of life. One study that examined health-related quality of life measures among adults with and without arthritis found that those with arthritis had higher mean numbers of days in the prior month when physical and mental health were not good (*27*). In this study, approximately one in four adults with arthritis reported ≥14 physically and ≥14 mentally unhealthy days during the past 30 days.

Evidence-based interventions (https://www.cdc.gov/arthritis/interventions/index.htm) have been reported to have a positive impact on arthritis outcomes (*22*,*23*); however, interventions are underused and require more widespread dissemination. Physical activity is a proven strategy for managing arthritis symptoms and many other chronic conditions (*22*). For instance, a meta-analysis of community-based physical activity interventions indicated that physical activity can decrease pain and improve function by approximately 40% (*22*). Although persons with arthritis report typical barriers to being physically active (e.g., lack of time and lack of enjoyment), arthritis presents specific barriers (e.g., pain, functional limitations, depression, and fear of falling and injury) (*28*). Similarly, in a meta-analysis of self-management education interventions, participants experienced improvements of 10%–20% in confidence and skills to manage their condition and reductions in pain, fatigue, and depression (*23*). However, as this and other analyses illustrate, self-management education interventions are underused by adults with arthritis; nationally, only about 11% report ever having taken a course (*19*).

Arthritis is a common comorbid condition that might complicate the management of other chronic conditions (e.g., obesity, coronary heart disease, and diabetes), increase the negative outcomes of these conditions, and reduce quality of life (*18*,*29*). The combination of arthritis and one of these chronic conditions has been associated with higher levels of physical inactivity (*30*–*32*). Moreover, arthritis also might hinder the ability of adults with prediabetes to engage in the level of physical activity recommended to prevent diabetes (*33*). Counseling persons with arthritis that physical activity can improve these outcomes (e.g., lower risk for diabetes) and improving availability of safe and effective physical activity programs in their local communities can be an effective strategy for reducing physical inactivity among these groups (e.g., adults with prediabetes or other comorbid conditions).

CDC funds arthritis programs in 12 state health departments and with national partners (e.g., Young Men’s Christian Association [YMCA] and National Recreation and Park Association) to disseminate evidence-based interventions in their communities (https://www.cdc.gov/arthritis/partners/funded-states.htm). State health departments, local community-based organizations, policymakers, and others can use the estimates at the state, territory, and county levels in this report to help identify local areas with need for evidence-based interventions. For example, physical activity programs such as EnhanceFitness, Walk With Ease, and Fit & Strong! (https://www.cdc.gov/arthritis/interventions/index.htm) could be disseminated to these areas. Several CDC-funded state health departments have been successful at reaching persons with arthritis with these evidence-based programs by partnering with YMCA of the USA and local parks and recreation departments.

## Limitations

The findings in this report are subject to at least seven limitations. First, arthritis was self-reported and not confirmed by a health care professional; however, this case definition has been shown to have sufficient sensitivity for public health surveillance (*34*). Second, because BRFSS is a cross-sectional survey, a causal relation between risk factors (e.g., obesity) and arthritis cannot be established, although robust evidence exists that links obesity to an increased risk for knee osteoarthritis (the most common form of arthritis) (*14*). Third, social desirability bias might have a role in certain self-reported characteristics, with underreporting of BMI (*35*) and overreporting of leisure-time physical activity (*36*). Fourth, the 2015 BRFSS median response rate was 47.2% and ranged from 33.9% to 61.1%, indicating potential nonresponse bias, although survey weights were applied to address this bias and improve external validity (*27*). Fifth, some of the morbidities potentially related to arthritis (e.g., physically or mentally unhealthy days) might be primarily affected by other conditions and thus might overestimate arthritis-specific impact. Sixth, the model used for county-level estimates did not account for complex sample design features, including potential geographic correlations between counties or states (i.e., observations for nearby counties and states might be clustered and therefore not independent). Finally, because county representativeness was not captured by BRFSS, model-based estimation other than direct survey estimation was used to generate prevalences at the county level. This approach has limitations that have been described elsewhere (*11*); however, the method has been tested through a comparison of model-based estimates with direct local survey estimates for certain other chronic conditions at the county level (*37*).

## Conclusion

In 2015, the number of adults with arthritis continued to increase, matched projections of prevalence, and exceeded projections for arthritis-attributable activity limitations at the state level (*38*). The findings in this report describe the prevalence and health-related characteristics of arthritis across the United States. The findings also highlight geographic variability in these estimates, including gaps in arthritis management. Public health professionals can use this information to better understand and target evidence-based nonpharmaceutical interventions, such as arthritis self-management education and physical activity. These interventions can decrease the impact of arthritis, which in turn might help adults with arthritis better manage comorbid conditions such as obesity, coronary heart disease, and diabetes. These estimates demonstrate the need to create links in clinical and community settings that can enhance health care provider counseling for physical activity and weight loss and facilitate referrals to self-management education and physical activity interventions to address arthritis and related comorbidities.
